# ZBRK1, a novel tumor suppressor, activates VHL gene transcription through formation of a complex with VHL and p300 in renal cancer

**DOI:** 10.18632/oncotarget.3134

**Published:** 2015-02-10

**Authors:** Ke Chen, Gan Yu, Kiranmai Gumireddy, Anping Li, Weimin Yao, Lu Gao, Shuliang Chen, Jun Hao, Ji Wang, Qihong Huang, Hua Xu, Zhangqun Ye

**Affiliations:** ^1^ Department of Urology, Tongji Hospital, Tongji Medical College, Huazhong University of Science and Technology, Wuhan, China; ^2^ Institute of Urology, Tongji Hospital, Tongji Medical College, Huazhong University of Science and Technology, Wuhan, China; ^3^ The Wistar Institute, Philadelphia, PA, USA; ^4^ Department of Cardiology, Institute of Cardiovascular Disease, Union Hospital, Tongji Medical College, Huazhong University of Science and Technology, Wuhan, China; ^5^ School of Basic Medical Sciences, Wuhan University, Wuhan, China; ^6^ Cardiovascular Research Institute of Wuhan University, Wuhan, China; ^7^ Department of Cell Death and Cancer Genetics, The Hormel Institute, University of Minnesota, Austin, MN, USA

**Keywords:** ZBRK1, VHL, p300, renal cancer, tumor suppressor

## Abstract

Inactivation or mutation of the VHL gene causes various tumors, including clear cell renal cell carcinoma (ccRCC). In the present study, we identified ZBRK1 as a novel VHL interacting protein by yeast two-hybrid screening, and found a single ZBRK1-binding site located in the VHL promoter region. Ectopic expression of ZBRK1 increases transcriptional activity of the VHL, whereas the depletion of endogenous ZBRK1 by shRNA leads to reduction of VHL expression. We also demonstrate that the inhibition of VEGF transcription by ZBRK1 overexpression is dependent on VHL/HIF pathway. Moreover, VHL is confirmed to serve as a bridge component for the association of ZBRK1 and p300, which leads to an increase in ZBRK1 transcriptional activity in the VHL promoter. We further provide striking evidences that ZBRK1 acts as a tumor suppressor in renal carcinoma by a variety of *in vitro* and *in vivo* assays, and ZBRK1 may represent a molecular marker to distinguish patients with ccRCC at high risk from those with a better survival prognosis. Taken together, these findings suggest that ZBRK1 suppresses renal cancer progression perhaps by regulating VHL expression.

## INTRODUCTION

Von Hippel–Lindau Protein (VHL) is a gatekeeper preventing renal cancer initiation [1–4], and mutations in VHL account for up to 70% of hereditary clear cell renal cell carcinoma (ccRCC) [5–7]. VHL has two domains, the α-domain that serves as elongin C/elongin B/Cul2/Rbx1 complex binding site and the β-domain plays a role in the substrate recognition [8]. Functions of VHL can be classified into two major types according to the dependence of hypoxia-inducible factor (HIF) [9], of which the best-documented is to target α subunit of HIF (HIF1α and HIF2α) for degradation. In this process, inactivation of VHL leads to up-regulation of HIF transcriptional activity and changes the expressions of HIF target genes with tumorigenicity [9]. Nevertheless, VHL can also functions independent of HIF, including maintenance of the primary cilium, extracellular matrix formation, apoptosis, and transcription modulations [9, 10]. Although accumulating evidences demonstrated the pivotal roles of VHL with multiple cellular functions, molecular partners of VHL still remain to be identified.

ZBRK1 was initially identified as a BRCA1 interaction protein by Y2H screen. ZBRK1 is a nuclear protein that contains a KRAB domain, eight C2H2 zinc fingers, and a C-terminal transcriptional repression domain (CTRD), which binds to a consensus sequence of GGGxxxCAGxxxTTT and functions as a transcriptional repressor [11]. At molecular and cellular levels, ZBRK1 regulates the expression of target genes with diverse functions. ZBRK1 was reported to interact with CtIP and BRCA1 and repress the expression of ANG1 and HMGA2 via ZBRK1 recognition sites in the promoter regions of these target genes [12, 13]. ZBRK1 also acts on MMP9 promoter to reduce MMP9 expression and thus inhibits metastasis of cervical carcinoma [14], and loss of ZBRK1 expression was found to increase KAP1 expression and promote metastasis and invasion [15]. Moreover, ZBRK1 interacts with ataxin-2 and activates SCA2 gene transcription [16]. In addition, low ZBRK1 expression was observed in many types of human cancers, including breast cancer, hepatocellular carcinomas, colon cancer and cervical cancer [14–18]. Furthermore, enhancement of ZBRK1 expression in HeLa cells inhibited the cell growth, invasion, and metastasis [14]. Thus, these results suggested that ZBRK1 functions as a transcriptional factor with tumor suppressor characters and plays a critical role in tumor development and progression.

In this study, we identified ZBRK1 as a novel VHL interacting protein. Besides, we demonstrated here that ZBRK1 forms an activator complex with p300 and VHL on ZBRK1 recognition site in VHL promoter, and the association of VHL and p300 is essential for the synergistical transcriptional activation of ZBRK1 in VHL promoter. Our data also showed that ZBRK1 over-expression led to an increase in VHL levels and a decrease in VEGF expression in ACHN cells. Furthermore, reduction of ZBRK1 expression was observed in ccRCC cells compared with its corresponding normal tissue, and ectopic expression of ZBRK1 inhibits cell proliferation, colony formation, and cell migration *in vitro* and suppresses carcinogenesis *in vivo*. The results from this study suggest that ZBRK1 plays a critical role in ccRCC progression by directly interacting with VHL and modulating VHL expression.

## RESULTS

### Identifacation of ZBRK1 as a novel VHL binding protein

**Figure 1:**
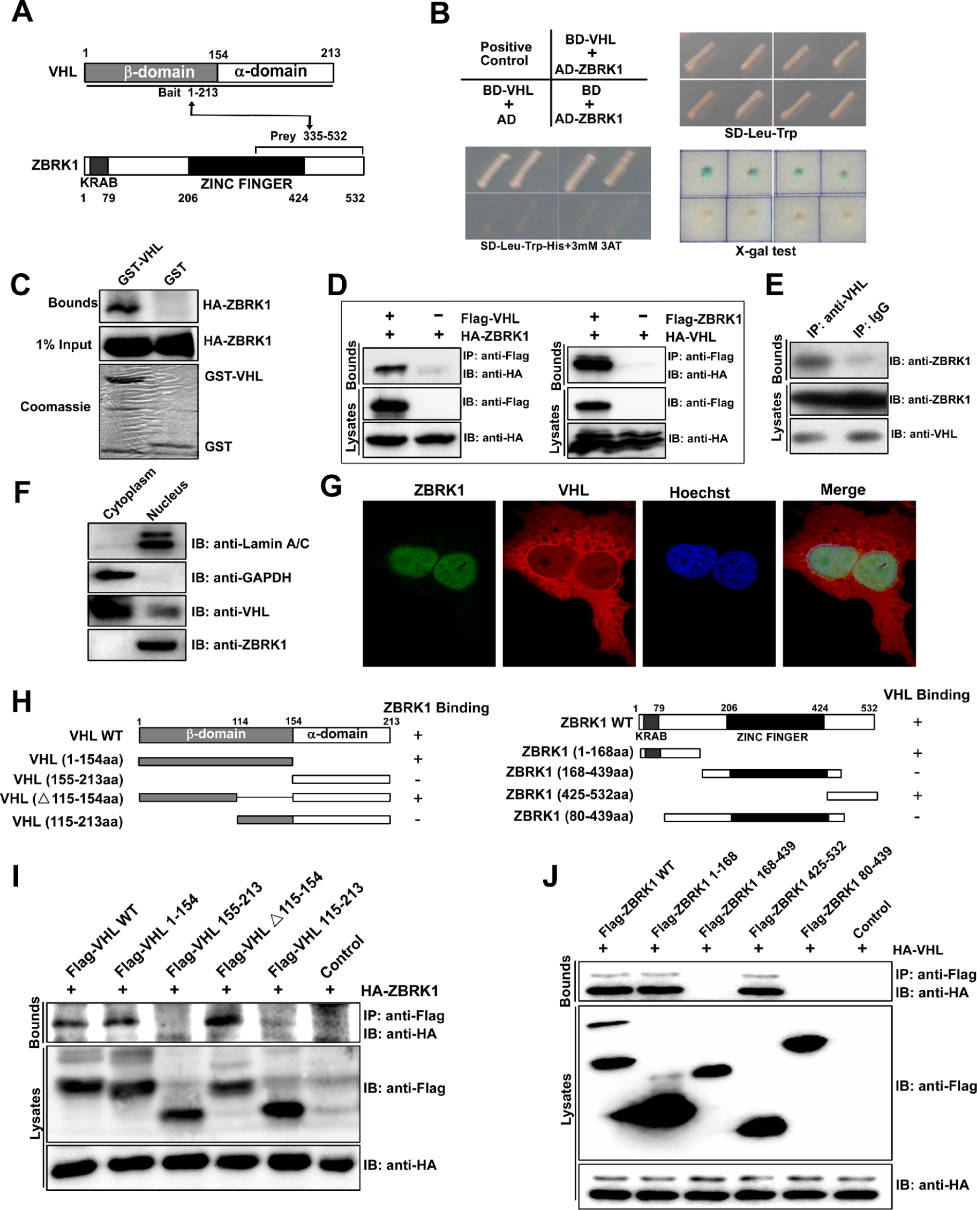
Identification of ZBRK1 as a VHL interacting protein **(A)** With VHL as bait, the yeast two-hybrid approach isolated a C-terminal fragment of ZBRK1 as prey. **(B)** ZBRK1 interacts with VHL in a yeast two-hybrid assay. Yeast AH109 cells were co-transformed with the indicated combinations of plasmids (left, top). The single yeast colonies containing these plasmids were grown on SD-Leu-Trp (left, bottom) agar plates and on SD-Leu-Trp-His with 25 mM 3AT (3-Amimo-1,2,4-Triazole) agar plates (right, top) and were tested by the X-Gal assay (right, bottom). Abbreviations: AD, pGADT7; BD, pGBKT7. **(C)** A direct interaction between GST-VHL and Flag-ZBRK1 proteins. The GST or GST-VHL purified from *E. coli* was incubated with Flag-ZBRK1-expressing HEK293T lysates and precipitated with glutathione-Sepharose. Precipitates were subjected to SDS-PAGE and examined by immunoblotting with anti-Flag (ZBRK1) antibody. Protein purities were confirmed by Coomassie Blue staining. **(D)** Co-IP of VHL and ZBRK1. (Left): HEK293T cells were transfected with the mammalian expression vectors Flag-VHL and/or HA-ZBRK1 as indicated. (Right): HEK293T cells were transfected with Flag-ZBRK1 and/or HA-VHL. The cell lysates were immunoprecipitated with anti-Flag and immunoblotted with anti-HA. **(E)** Endogenous interaction between ZBRK1 and VHL. ACHN cell lysates were immunoprecipitated (IP) with a control antibody (rabbit IgG) or an anti-VHL antibody and analyzed by immunoblotting (IB) with anti-ZBRK1. **(F)** Western blot analysis confirmed nuclear expression of both ZBRK1 and VHL proteins. Lamin A/C and GAPDH were used as internal controls for the nuclear and cytoplasmic extracts, respectively. **(G)** Co-localization of VHL with ZBRK1 in the nucleus imaged by confocal microscopy. Caki-1 cells were fixed, permeabilized, and stained with the mixture of two primary antibodies for overnight at 4°C. Then, the cells were subjected to the mixture of two secondary antibodies (Cy3-conjugated against mouse and FITC-conjugated against rabbit). Finally, the cells were treated with Hoechst 33258 for the nucleus staining and observed by confocal microscope. **(H)** Domain structure and deletion constructs of VHL (left) and ZBRK1 (right). Numbers refer to amino acids. **(I)** Mapping of the ZBRK1-binding region of VHL. HEK293T cells were transiently transfected with HA-ZBRK1 along with various FLAG-tagged VHL deletion mutants as indicated. The cell lysates were immunoprecipitated with anti-Flag antibody and immunoblotted with anti-HA antibody. **(J)** Mapping of the VHL binding region of ZBRK1. HEK293T cells were transiently transfected with HA-VHL along with various FLAG-tagged ZBRK1 deletion mutants as indicated. The cell lysates were immunoprecipitated with anti-Flag antibody and immunoblotted with anti-HA antibody.

In order to gain further insights into the function of VHL, we performed an Y2H screen with VHL as bait protein. From this study, one encodes for part of the C-terminal region of ZBRK1 were identified as positive prey clones by independent Y2H experiments and sequencing analysis (Figure 1A). To verify the potential interaction between VHL and ZBRK1, we co-transformed AH109 yeast cells with BD-VHL and AD-ZBRK1 (Figure 1B, left, top), and observed that co-expression of VHL and ZBRK1 significantly activated reporter gene (Figure 1B, bottom). To further tested the protein-protein interaction of ZBRK1 and VHL, GST-pulldown assay and co-immunoprecipitation assay were performed. As expected, we found that ZBRK1 interacts directly with VHL *in vitro* and *in vivo* (Figure 1C, 1D). Endogenous protein-protein interaction of ZBRK1 and VHL was also observed in ACHN cells (Figure 1E). In addition, western blot analysis revealed that VHL existed in both the cytoplasm and nucleus, and ZBRK1 was only detected in the nucleus (Figure 1F). In accord with this, immunofluorescence analysis showed that both ZBRK1 and VHL were co-localized in the nucleus, although the majority of VHL was expressed in the cytoplasm (Figure 1G). Thus, these results demonstrated that ZBRK1 interacts with VHL in the nucleus.

To identify the critical protein domains for VHL binding to ZBRK1, we generated a series of truncated Flag-tagged VHL constructs (Figure 1H, left) and co-transfected VHL deletion mutants with HA-ZBRK1 followed by co-IP. Two VHL mutants, Flag-VHL 1–154 aa and Flag-VHL Δ115–154 aa, were found to interact with ZBRK1 (Figure 1I), indicating that the N-terminal region (1–114 aa) in VHL β domain is critical for the binding to ZBRK1. Using a series of deletion mutants of ZBRK1 (Figure 1G, right), we further identified that both KRAB and CTRD domains were capable to interact with VHL (Figure 1J).

### Loss of ZBRK1 expression is associated with poor prognosis in patients with renal cancer and contributed to the renal cancer progression

**Figure 2:**
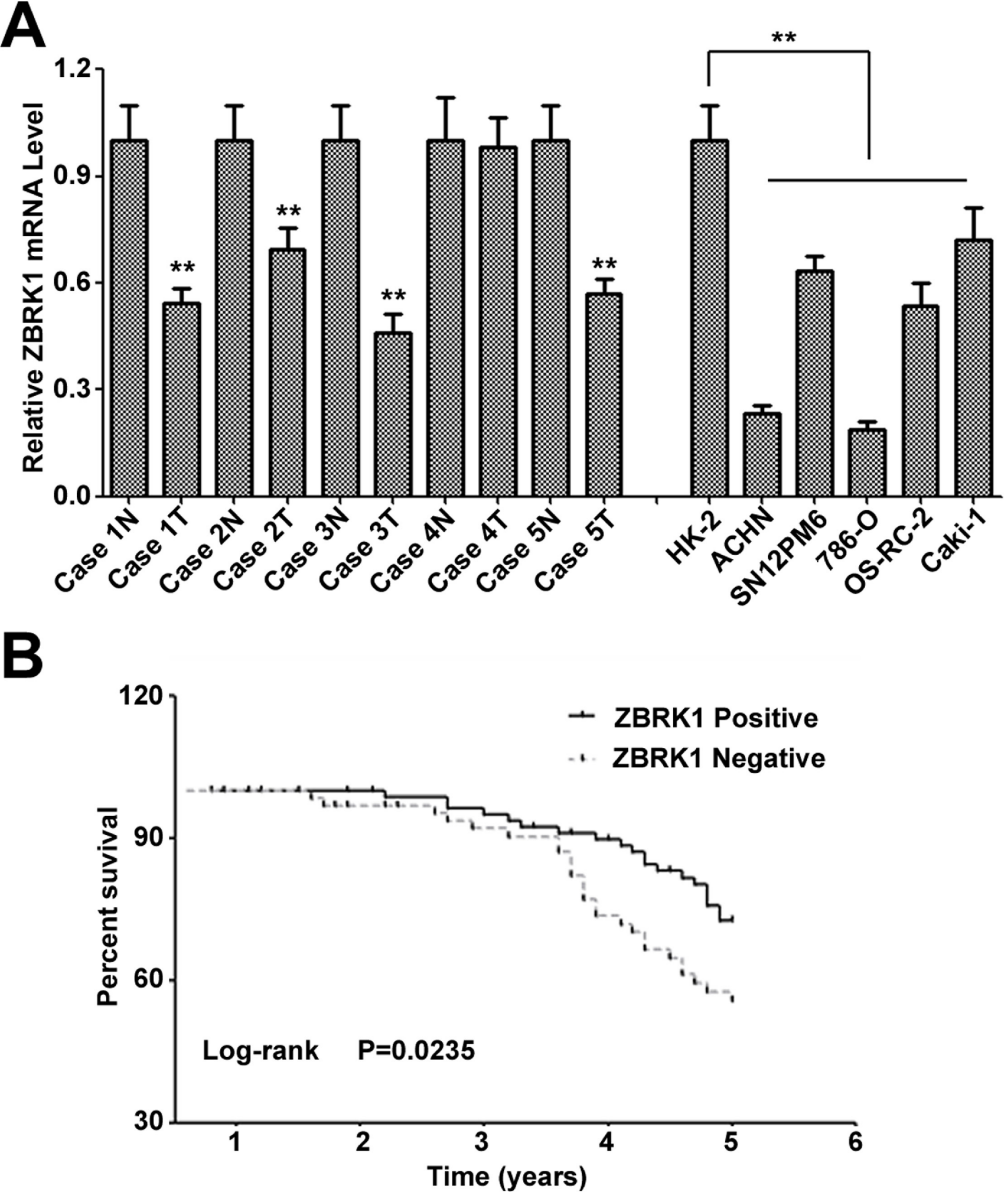
Loss of ZBRK1 expression is associated with poor prognosis in patients with renal cancer and contributed to the renal cancer progression **(A)** Relative expression of ZBRK1 mRNA expression levels were evaluated by real-time PCR in non-tumorigenic and renal cancer cell lines and paired case specimens. **(B)** Kaplan-Meier overall survival curves for renal cancer patients. The ZBRK1 with negative expression correlated with a low overall survival rate. ** indicates significant differences, *P* < 0.01.

To determine the roles of ZBRK1 expression on renal cancer development and progression, we examined the mRNA level of ZBRK1 in 5 paired renal cancer tissue and tumor adjacent renal tissue specimens, and in a panel of 6 renal cell lines including 5 cancerous cell lines (ACHN, 786-O, OS-RC-2, CaKi-1 and SN12PM6) and control cell line HK-2 (human kidney proximal tubular epithelial cell) using quantitative PCR analysis. It revealed that 5 of 5 renal cancer cell lines and 4 of 5 renal cancer specimens manifest as noticeably down-regulation of ZBRK1 mRNA as compared with the corresponding controls (Figure 2A), suggesting that reduction of ZBRK1 expression may be involved in renal cancer development and progression. We thus investigated the clinical relevance of ZBRK1 in paired renal cancer specimens. It showed that,, in the primary renal cancer tissue specimens, the level of ZBRK1 expression can be divided into two categories: negative and positive. No remarkably difference of ZBRK1 expression was found in the distribution according to sex and age. However, we observed significant difference in the distribution of the patients according to pathologic grade (*P* = 0.042), clinic stage (*P* = 0.0228) and lymph node metastasis (*P* < 0.01) (Table 1). Kaplan-Meier curves and the log-rank test also showed significance of decreased ZBRK1 with over survival (*P* = 0.0235) (Figure 2B). Therefore, these results indicated that decreased ZBRK1 expression plays a critical role in renal cancer development and progression and is a valuable biomarker for this disease.

**Table 1:** Clinical characteristics and outcome of 155 renal clear cell cancer patients according to ZBRK1 gene expression status

Characteristic	Total (*n* = 155)	ZBRK1		*P*
		Positive	Negative	
**Sex**				
** Men**	108	38	70	0.693
** Women**	47	15	32	
**Age (years)**				
** Mean (SD)**	62.8 (14.7)	61.4 (13.2)	63.7 (16.1)	
**Grade**				
** 1–2**	130	48	82	0.042
** 3–4**	25	4	21	
**Stage**				
** T_1–2_**	138	44	94	0.0228
** T_3–4_**	17	3	14	
**Lymph node metastasis**				
** N_0_**	150	51	99	
** N_1–2_**	5	1	4	<0.01

**NOTE:** Two-tailed fisher's exact test was done with the SPSS software program to determine the statistical significance of the level of expression of ZBRK1 in different tissue specimens.

### ZBRK1 inhibits cell growth, tube formation, migration and invasion in renal cancer

**Figure 3:**
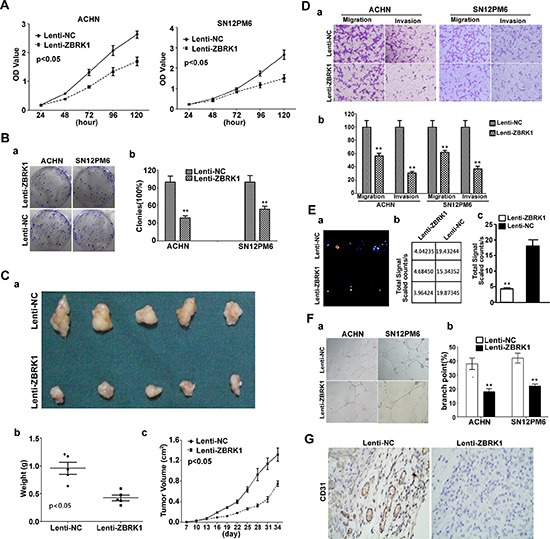
ZBRK1 inhibits cell growth, tube formation, migration and invasion in renal cancer **(A)** CCK-8 kit was utilized to quantify cell viability at each time point. Data are plotted as the mean ± SEM of 3 independent experiments. **(B)** a’. Representative photographs of cell culture plates following staining for colony formation of ACHN and SN12PM6 cell. b’. Number of colonies was quantified. **(C)** a’. Photographs of tumors excised 34 days after inoculation of stably transfected cells into nude mice. b’. Tumor weight of each nude mouse at the end of 34 days. c’. Mean tumor volume measured by caliper on the indicated days. **(D)** a’. Migration and invasion assay for renal cancer cells. Representative photographs were taken at × 200 magnification. b’. Number of migrated and invaded cells were quantified in 4 random images from each treatment group. Results are the mean ± SEM from 3 independent experiments plotted as percent (%) migrating and invading cells relative to Lenti-NC treatment. **indicates significant differences, *P* < 0.01. **(E)** a’. Representative bioluminescent images of lungs of nude mice at the 30th days after IV. injection of renal cancer cell. b’ the precise data of fluorescence signal from captured bioluminescence images. c’. Quantification analysis of fluorescence signal from captured bioluminescence images. **indicates significant differences, *P* < 0.01. **(F)** a’. Tube formation of HUVECs was determined by assaying the numbers of branch nodes after 6 h of culture under a phase contrast microscope. HUVECs were cultured in the following media: CM of ACHN and SN12PM6 cells transfected with negative control and CM of ACHN and SN12PM6 cells infected with Lenti-ZBRK1. b’, Number of branch point was quantified. **(G)** Endogenous CD31 expression in xenograft tumors derived from ACHN cells expressing Lenti-NC and Lenti-ZBRK1 using anti-CD31. Signals were detected with horseradish peroxidase-conjugated secondary antibody (brown).

To further investigate the roles of ZBRK1 in the development of renal cancer, we over-expressed ZBRK1 in ACHN and SN12PM6 cells by lentiviral vector, and examined the effect of ZBRK1 on the cell proliferation and colony formation. Our data showed that over-expression of ZBRK1 in ACHN and SN12PM6 cells significantly decreased cell viability and reduced ability of colony formation of these cells *in vitro* (Figure 3A and 3B). While the Caki-1 cell viability and ability of colony formation steadily increased following stable RNA knockdown by lentiviral transfer of ZBRK1-specific shRNA (Supplementary Figure S1A, S1B). We also found that over-expression of ZBRK1 inhibited *in vivo* tumor growth in xenograft models with statistically significance (Figure 3C). Next, in order to test the effects of ZBRK1 on cancer cell migration, ACHN and SN12PM6 cells were infected with Lenti-ZBRK1 or Lenti-NC and allowed to migrate through a transwell membrane into complete media. Compared with the negative control, over-expression of ZBRK1 inhibited cell migration by 43% and 38% reduction in migratory ACHN and SN12PM6 respectively (Figure 3D). ZBRK1 over-expression also significantly reduced invasion capability of ACHN and SN12PM6 cells. As shown in Figure 3D, approximately 69% and 61% reduction of invading ACHN and SN12PM6 cells were observed in the Lenti-ZBRK1 infected cell when compared with the cells infected with empty vector control. The reverse effect was shown in the Caki-1 cells as ZBRK1 was down-regulated with the lentiviral transfer of ZBRK1-specific shRNA (Supplementary Figure S1C). In IV. Injection assay with bioluminiscence imaging, we noticed that fluorescence signal in Lenti-ZBRK1 group was significantly weaker than Lenti-NC group, suggesting that less metastasis formed in lung after ZBRK1 over-expression (Figure 3E).

We further determined the angiogenic activity of conditioned media of cells infected with Lenti-ZBRK1. We found that tube formation by activated HUVECs was achieved by the conditioned media (CM) of SN12PM6 and ACHN cells infected with Lenti-ZBRK1 or CM of SN12PM6 and ACHN cells infected with Lenti-NC. The angiogenic activity of CM lost by ectopic over-expression of ZBRK1 (Figure 3F). To further characterize the *in vivo* effects of ZBRK1 on angiogenesis, ACHN tumors were evaluated for blood vessel density. The tumors were labeled with CD31, an endothelial cell–specific marker. Immunohistochemistry results showed that blood vessel quantification reduced dramatically in the ACHN tumors over-expressing ZBRK1 (Figure 3G). As expected, the angiogenic activity was increased (Supplementary Figure S1D) in Caki-1 cells stably expressing the knockdown constructs (lentiviral transfer of ZBRK1-specific shRNA). All of the above data indicated that ZBRK1 inhibits renal tumor angiogenesis.

Taken together, these results suggested that ZBRK1 expression inversely correlates with the malignancy of cancerous cells and ZBRK1 acts as a tumor suppressor to inhibit cell growth, tube formation, migration and invasion in renal cancer.

### ZBRK1 inhibits the transcription of VEGF through VHL/HIF pathway

**Figure 4:**
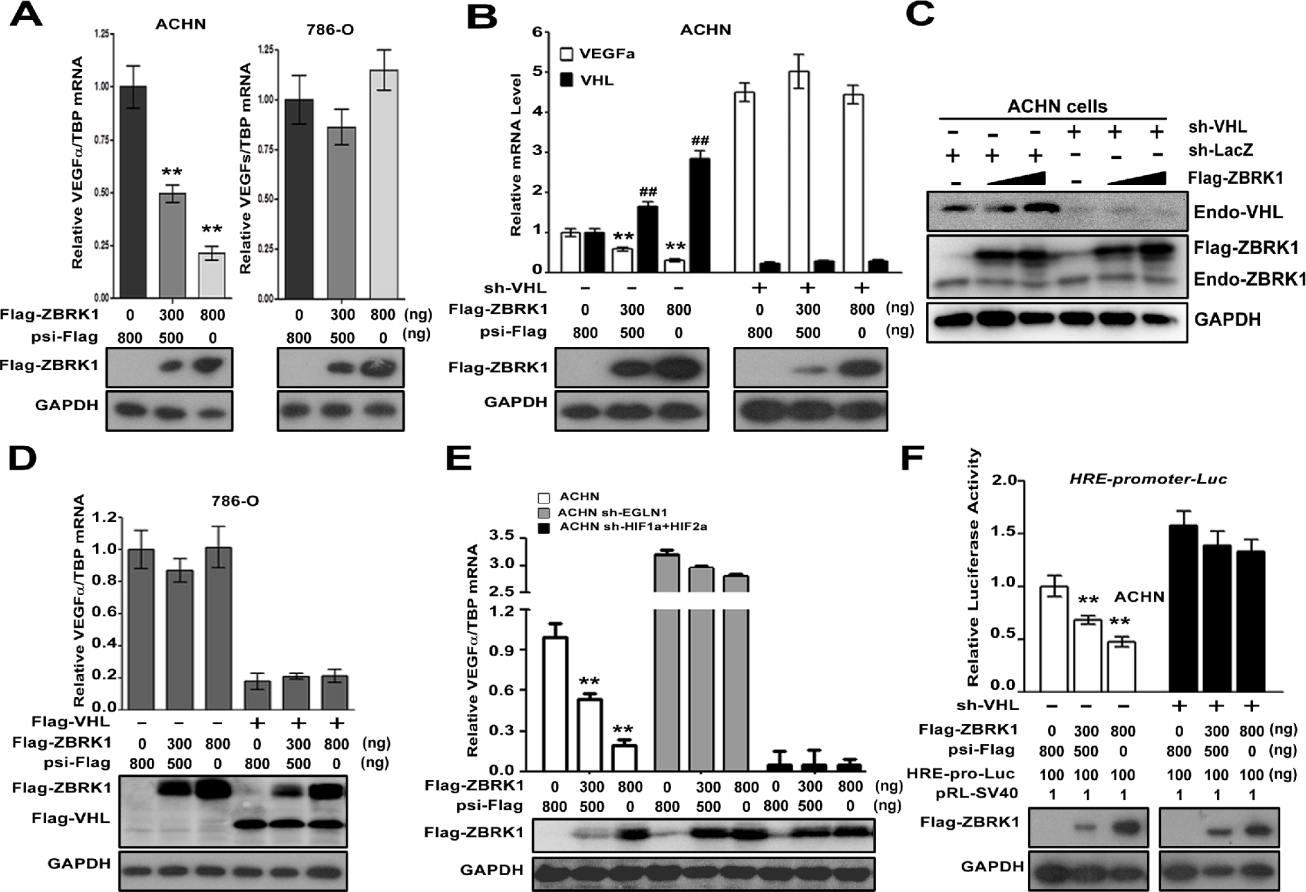
ZBRK1 inhibits the transcription of VEGF through the VHL/HIF pathway **(A)** A gradual increase in ZBRK1 inhibits the transcriptional of endogenous VEGF in ACHN cells (left), but not in 786-O cells (right). ACHN cells or 786-O cells were transfected with increasing amount of Flag-ZBRK1. After transfection for 48 h, total RNAs were prepared and Real-time PCR was carried out using specific primers for VEGFα and TBP (inner control). Expression was normalized to cells transduced with the control vector (psi-Flag, Ctrl), data are plotted as the mean ± SD of 3 independent experiments. **p < 0.01 vs. Ctrl. **(B)** The effect of ZBRK1 on the expression of VEGF is mediated by VHL. ACHN cells stably expressing VHL-shRNA were transfected with increasing amount of Flag-ZBRK1. Expression was normalized to cells transduced with the control vector, data are plotted as the mean ± SD of 3 independent experiments. **p < 0.01 vs. Ctrl. **(C)** The protein level of VHL was increased when ZBRK1 was over-expressed. ACHN cells or ACHN cells stably expressing *VHL*-shRNA were transiently transfected with increasing amount of Flag-ZBRK1. Cell lysates were subjected to SDS-PAGE followed by immunoblotting with anti-ZBRK1, anti-VHL, and anti-GAPDH antibody. **(D)** ZBRK1 has no inhibitory effect on the expression of VEGF in the 786-O cells which rescued VHL expression. 786-O cells stably expressing Flag-VHL were transfected with increasing amount of Flag-ZBRK1. **p < 0.01 vs. Ctrl. **(E)** The effect of ZBRK1 on the expression of VEGF is mediated by HIF pathway. ACHN cells stably expressing HIFα-shRNA or EGLN1-shRNA were transfected with increasing amount of Flag-ZBRK1. **p < 0.01 vs. Ctrl. **(F)** VHL is required for ZBRK1 mediated inhibition of HRE-promoter-driven luciferase activity. ACHN cells stably expressing LacZ-shRNA or VHL-shRNA were transiently transfected with 6xHRE-promoter-driven luciferase reporter genes and pRL-SV40 (inner control, SV40 promoter with Renilla luciferase reporter gene) along with increasing amount of Flag-ZBRK1. After transfection for 36 H, luciferase activities were measured and normalized with inner control. **p < 0.01 vs. Ctrl.

We next sought to determine the consequences of ZBRK1 binds to VHL. Previous studies showed that VHL represses the transcription of VEGF through both HIF dependent and independent pathways [9, 19]. In order to test whether ZBRK1 regulates the expression of VEGF, Flag-ZBRK1 with ever-increasing concentrations was transfected into ACHN cells. Consistently, Real-time PCR assays showed that ZBRK1 inhibited the expression of endogenous VEGFα in a dose-dependent manner in ACHN cells (Figure 4A, left). Interestingly, we noticed that the expression of Flag-ZBRK1 did not lead to obvious changes of VEGFα expression in VHL^−/−^ 786-O cells (Figure 4A, right). Besides, when ZBRK1 was transiently over-expressed, protein level of VEGFα was decreased in a dose-dependent manner in ACHN cells but not in 786-O cells (Supplementary Figure S2). We thus speculated that VHL is essential for ZBRK1-blocked the expression of VEGF. To test this hypothesis, ACHN cells were infected with VHL shRNA prior to transfection with Flag-ZBRK1. We found that the inhibitory effect of ZBRK1 on VEGF expression significantly decreased (Figure 4B), suggesting that the regulatory effect of ZBRK1 on VEGF expression relies on the presence of VHL.

Besides, we noticed that over-expression of ZBRK1 increased VHL mRNA and protein levels in ACHN cells (Figure 4B and 4C). To further determine whether the transcription of VHL is necessary for ZBRK1 down-regulates VEGF expression, 786-O cells (VHL^−/−^) were treated with lentiviral plasmid expressing Flag-VHL prior to transfected with Flag-ZBRK. The results indicated that ZBRK1 has no inhibitory effect on VEGF expression (Figure 4D). This finding suggested that ZBRK1 inhibits the expression of VEGF dependent on up-regulate VHL transcription.

To investigate whether ZBRK1 suppresses the expression of VEGF is HIF-dependent, we established a series of ACHN cell lines using stable RNA knockdown by lentiviral transfer of *EGLN1*-specific shRNA, *HIF1α*-specific shRNA, or/and *HIF2α*-specific shRNA. As expected, EGLN1, HIF1*α* or HIF2*α* mRNA levels were reduced in cells stably expressing the corresponding knockdown constructs (Supplementary Figure S3A, S3B and S3C). VEGF mRNA levels were reduced in cells expressing *HIF1α*-specific shRNA or/and *HIF2α*-specific shRNA, whereas the expression of VEGF was increased in cells expressing *EGLN1*-specific shRNA (Supplementary Figure S3D). These results indicated an effectiveness of these shRNA. Next, qPCR analysis showed that ZBRK1 inhibited the expression of *VEGF* in ACHN cells stably expressing a control nonspecific (LacZ) shRNA, whereas the inhibitory effect of VEGF expression by ZBRK1 was remarkably attenuated in ACHN cells stably expressing *EGLN1*-specific shRNA, or *HIF1α*-shRNA and *HIF2α*-shRNA (Figure 4E). It was reported that HRE-containing genes are well-known downstream target gene of VHL/HIF pathway [9]. We thus utilized a HRE-luc reporter system to examine the role of HIF on ZBRK1-dependent inhibitory effects of VEGF. Expression of ZBRK1 inhibited the transcriptional activation of the HRE-luc reporter gene in ACHN cells expressing control shRNA, whereas HRE-luc activity was unaffected in ACHN cells stably expressing *EGLN1*-specific shRNA, or *HIF1α*-shRNA and *HIF2α*-shRNA (Supplementary Figure S3E). These results suggested that ZBRK1 inhibits the expression of VEGF dependent on HIF.

To investigate the role of VHL on ZBRK1-dependent inhibition of VEGF transcription, we established ACHN cells with stable expression of LacZ-shRNA or VHL-shRNA, and then transiently transfected these engineered cells with increasing concentration of Flag-ZBRK1 together with HRE-promoter-driven luciferase. As expected, expression of ZBRK1 inhibited the transcriptional activation of the HRE-luc reporter gene in ACHN cells, which was remarkable attenuated in ACHN cells stably expressing VHL-shRNA (Figure 4F). To examine whether ZBRK1 is associated with the VEGFα promoter, we performed a chromatin immunoprecipitation assay focusing on five fragments in the VEGFα promoter. As shown in Supplementary Figure S3F, all of the DNA fragments in the VEGFα promoter were not enriched during immunoprecipitation with anti-ZBRK1 in ACHN cells. However, DNA fragments in GADD45 Intron 3 containing a ZBRK1 recognition sequence was highly enriched (Supplementary Figure S3F). Together with the above results, we reasoned that ZBRK1 inhibits the transcription of VEGF dependent on VHL/HIF pathway indirectly.

### VHL gene is a ZBRK1 target gene

**Figure 5:**
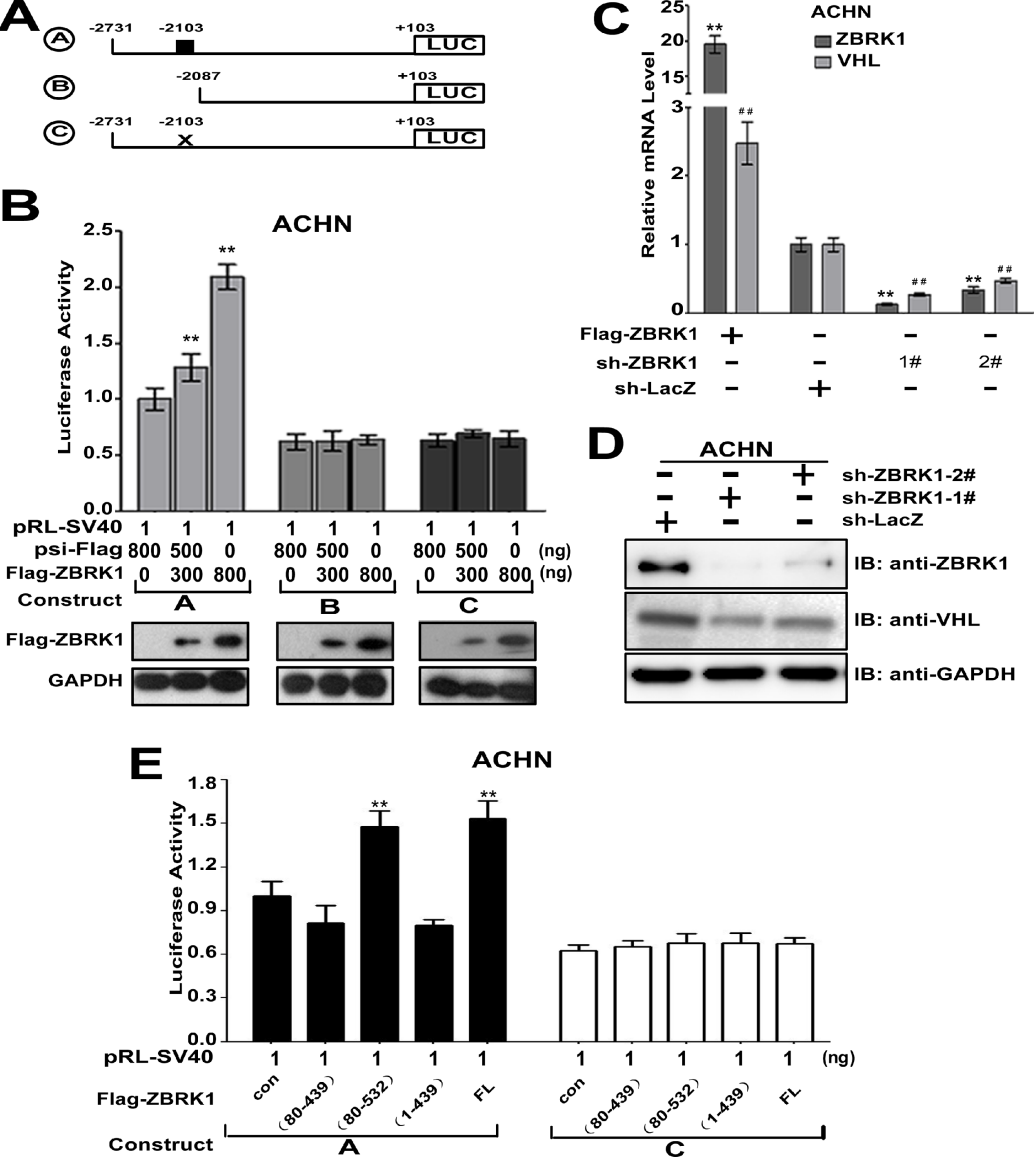
VHL gene is a ZBRK1 target gene **(A)** Schematics of luciferase reporter constructs A–B containing the VHL promoter with or without ZBRK1 binding motifs. The putative ZBRK1 recognition sequence is indicated by a small black box. A, −2731/+103; B, −2087/+103 from the start codon. C, the putative ZBRK1 recognition sequence is mutated. **(B)** The ZBRK1 recognition sequence in the VHL promoter plays an important positive role. ACHN cells were transfected with relevant luciferase reporter constructs A−C and pRL-SV40 along with increasing amount of Flag-ZBRK1, respectively. After transfection for 36 h, the relative activities of various VHL reporter constructs were analyzed, luciferase activities were measured and normalized with control (psi-Flag). Means ± standard deviations for *n* = 3 are shown. **p < 0.01 vs. Ctrl. **(C)** ZBRK1 expression level is positive associated with the VHL transcript level. Total RNAs were extracted from ACHN cells (~10^6^) expressing Flag-ZBRK1 or ZBRK1 RNAi plasmids [1#, 2#, and control vector] respectively, followed by qPCR using specific primers for ZBRK1, VHL and TBP (inner control), expression was normalized to cells transduced with the control vector (Ctrl), data are plotted as the mean ± SD of 3 independent experiments. **p < 0.01 vs. Ctrl/ZBRK1, ^##^*p* < 0.01 vs. Ctrl/VHL. **(D)** The protein level of VHL was decreased when ZBRK1 was knockdown by shRNA. **(E)** The CTRD domain of ZBRK1 is necessary in activating the VHL promoter activity. ACHN cells were transfected with relevant luciferase reporter constructs A or C and pRL-SV40 along with serial deletion mutants of Flag-tagged ZBRK1. The luciferase activities were measured and normalized with control. Means ± standard deviations for *n* = 3 are shown. **p < 0.01 vs. Ctrl.

We performed a VHL promoter analysis using ZBRK1 recognition site (GGGxxxCAGxxxTTT) [11]. A putative ZBRK1-binding motif (−2103/−2089) was identified in the VHL promoter. Thus, we cloned a series of VHL promoter with or without potential ZBRK1 binding motif in luciferase plasmid reporter. As shown in Figure 5A and 5B, ZBRK1 induced the luciferase activity in ACHN cells transfected with construct A, but not B or C, suggesting that the region −2103/−2089 represents a ZBRK1 recognition site involved in transcriptional regulation of VHL by ZBRK1. To further validate this, we used RNA interference to block ZBRK1 expression in ACHN cells. RT-qPCR results showed that the VHL expression levels in ZBRK1-depleted cells were dramatically reduced in comparison with the control cells (Figure 5C). Western blot analysis on the endogenous expression of ZBRK1 and VHL also confirmed that VHL was decreased in the ZBRK1 knockdown cells (Figure 5D). Given that ZBRK1 generally functions as a transcriptional repressor through association with co-repressor proteins, including KAP1 and BRCA1 [11, 12, 20, 21], we sought to determine whether ZBRK1 regulates the expression of VHL dependent on KAP1 and BRCA1. Treatment of ACHN cells by lentiviral transfer of *KAP1*-specific shRNA or *BRCA1*-specific shRNA depleted endogenous KAP1 or BRCA1, respectively (Supplementary Figure S4A and B). The activation effect of ZBRK1 on VHL expression was irrelevant to KAP1 or BRCA1 expression levels, respectively (Supplementary Figure S4C), suggesting that ZBRK1 activates VHL transcription independent of KAP1 and BRCA1. Considering that both KRAB and CTRD domains were capable to interact with VHL (Figure 1I), we examined whether the transcriptional activation of the VHL by ZBRK1 is dependent on KRAB or/and CTRD domain of ZBRK1. As shown, over-expression of ZBRK1 WT and ZBRK1 (80–532) significantly induced the luciferase activity in ACHN cells transfected with construct A, but not C. In contrast, transfection of cells with ZBRK1 (1–439) and ZBRK1 (80–439) failed to induced the luciferase activity of VHL promoter (Figure 5E). This result suggested that transactivational effects of ZBRK1 require its CTRD domains. Thus, our results suggested that ZBRK1 can activate VHL promoter activity through the ZBRK1-binding motif at −2103/−2089 in VHL promoter region.

### ZBRK1, VHL, and p300 form an activator complex on VHL promoter

Previous studies indicated that VHL recruit p300 when acts as a cofactor of p53 [22], we thus speculated whether VHL can mediate the interaction of ZBRK1 between p300.

**Figure 6:**
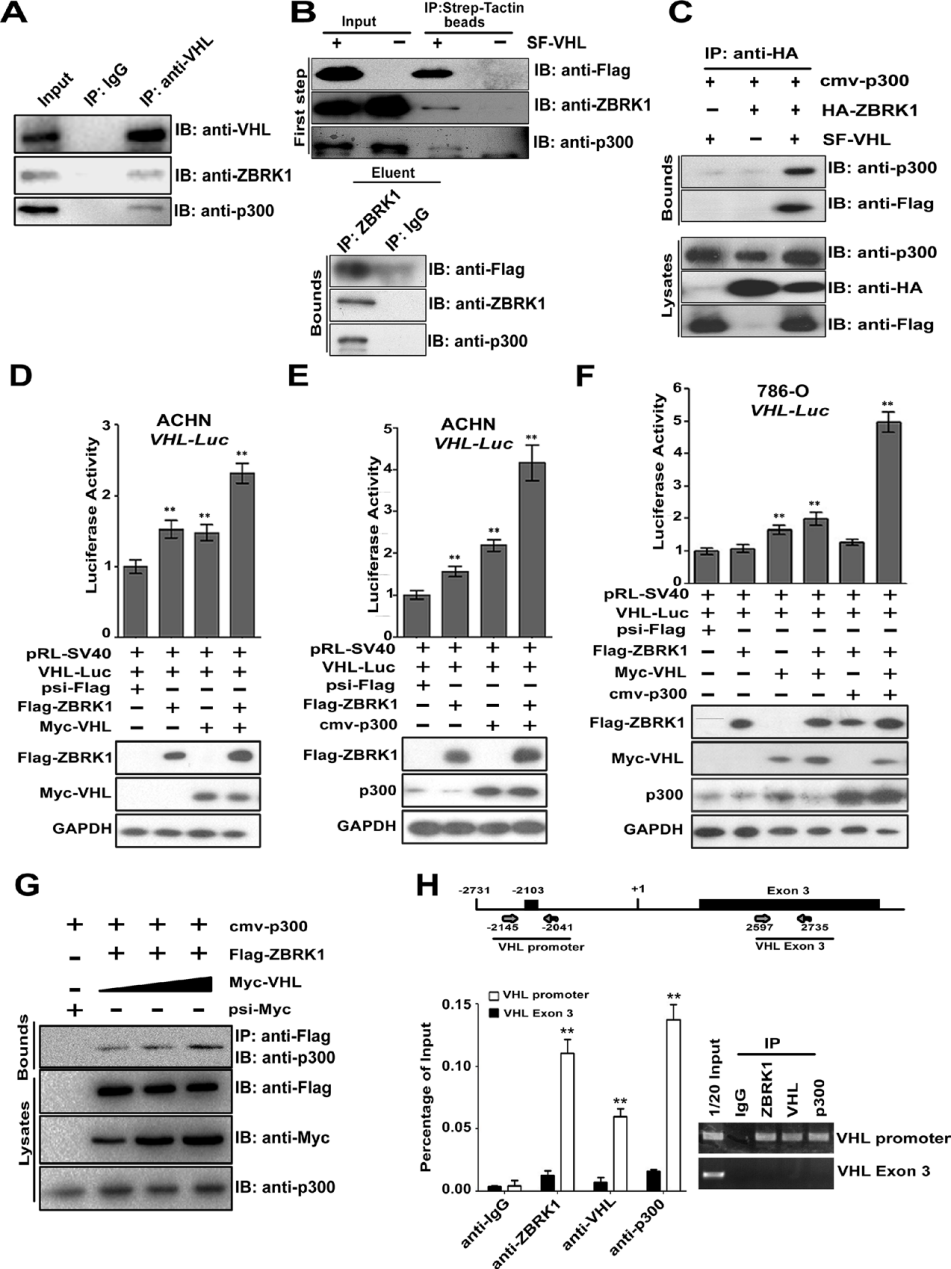
ZBRK1, VHL, and p300 form an activator complex co-activates VHL expression via a ZBRK1 recognition element in the VHL promoter **(A)** VHL interacts with ZBRK1 and p300 *in vivo*. ACHN cells (~2 × 10^7^) were collected for co-immunoprecipitation with anti-VHL, followed by probing with anti-VHL, anti-ZBRK1, and anti-p300. **(B)** p300 is a component in the VHL/ZBRK1 protein complex. ACHN cells (~6 × 10^7^) expressing SF-tagged (Strep II tag and Flag tag)-VHL were collected and lysed for Two-TAP scheme. In the first step, proteins associated with VHL were eluted from Strep-Tactin beads under native conditions with desthiobiotin, followed by immunoblotting with anti-Flag, anti-ZBRK1, and anti-p300 antibodies. In the second step, the eluent from Strep-Tactin beads were immunoprecipitated with anti-ZBRK1 and immunoblotted with anti-Flag and p300 antibodies. **(C)** VHL mediates the interaction between ZBRK1 and p300. 786-O cells (~10^7^) expressing p300, HA-ZBRK1, and/or SF-VHL were collected for co-immunoprecipitation with anti-HA, followed by probing with anti-Flag, and anti-p300. **(D)** VHL enhances the transcriptional activity of ZBRK1 in VHL promoter. ACHN cells were transiently transfected with different combinations of expression vectors for pRL-SV40, Flag-ZBRK1, and Myc-VHL along with VHL-promoter driven luciferase reporter gene, luciferase activities were measured and normalized with inner control. **p < 0.01 vs. psi-Flag. **(E)** p300 further increases ZBRK1-mediated transcriptional activity on VHL promoter. ACHN cells were transiently transfected with different combinations of expression vectors for pRL-SV40, Flag-ZBRK1, Myc-VHL, and/or p300 along with VHL-promoter driven luciferase reporter gene, Luciferase activities were measured and normalized with inner control. **p < 0.01 vs. psi-Flag. **(F)** p300 functions as a ZBRK1 co-activator through VHL. 786- O cells were transiently transfected with different combinations of expression vectors for pRL-SV40, Flag-ZBRK1, p300, and/or Myc-VHL along with VHL-promoter driven luciferase reporter gene. After transfection for 36 h, cells were lyzed, the relative luciferase activities were measured and normalized with inner control. **p < 0.01 vs. psi-Flag. **(G)** VHL helps the ZBRK1-p300 interaction. HEK293T cells were co-transfected with Flag-ZBRK1, cmv-p300, and increasing amount of Myc-VHL. Cell lysates were precipitated with anti-Flag antibody and immunoblotted with anti-p300 antibody. **(H)** ZBRK1, VHL, and p300 form a complex binds to the VHL promoter. ChIP analysis on a 105 bp fragment around a ZBRK1 binding site in VHL promoter (−2145/−2041) to detect the association of ZBRK1, VHL, and p300 in ACHN cells. The cells were lyzed and chromatin DNA was immunoprecipitated with anti-ZBRK1, anti-VHL, or anti-p300, validated by qPCR using primers encompassing the predicted ZBS and normalized to control DNA (a PCR product of +2597/+2735 located in Exon 3 of the VHL gene). Means ± standard deviations for *n* = 3 are shown. **p < 0.01 vs. anti-IgG/VHL promoter.

p300 and ZBRK1 were found to be associated with VHL in ACHN cells (Figure 6A), suggesting these proteins form a complex in cells. This finding was further verified by assay employed a tandem affinity purification (TAP). As shown in Figure 6B (top), ZBRK1 and p300 were detached after incubated with Strep-Tactin beads in the first affinity purification. After elution of proteins associated with VHL from beads with desthiobiotin, immunoprecipitation was performed using anti-ZBRK1 antibody. SF-VHL and p300 were found to be co-precipitated efficiently with ZBRK (Figure 6B, bottom). These results suggest the interaction of ZBRK1 and VHL forms a complex with p300. We also tested whether ZBRK1 associates with p300 in the presence or absence of VHL. p300 was found to be associated with ZBRK1 only in the presence of VHL (Figure 6C), indicating that ZBRK1 associates with p300 through its interaction with VHL.

We further examined the effects of p300 and VHL on the enhancement of ZBRK1 transcriptional activity. ACHN cells were transiently transfected with different combinations of ZBRK1 and/or VHL along with VHL-promoter driven luciferase reporter and pRL-SV40 (inner control, SV40 promoter with Renilla luciferase reporter gene). As expected, VHL over-expression led to an increase in the VHL promoter activity (Figure 6D). Moreover, p300 further enhanced ZBRK1-mediated VHL promoter activity (Figure 6E). Furthermore, similar results were obtained in Caki-1 cells (Supplementary Figure S4D, S4E). To further examine that ZBRK1 and VHL along with p300 forming a complex bound to the VHL promoter regulating VHL gene transcription, we transiently transfected 786-O cells with different combinations of ZBRK1, VHL, p300, VHL-promoter-driven luciferase reporter gene, and pRL-SV40, respectively, as indicated. ZBRK1 and/or p300 over-expression led to an increase in the VHL promoter activity only when VHL was transfected (Figure 6F). Given that significant elevation of VHL promoter activity when VHL was transfected alone, we sought to determine whether VHL promotes the ZBRK1-p300 interaction of VHL and p300. As expected, when VHL was transiently over-expressed, it helped the ZBRK1–p300 interaction in a dose-dependent manner (Figure 6G). Besides, qPCR analysis showed that ZBRK1 activated the expression of VHL in ACHN cells stably expressing a control nonspecific (LacZ) shRNA, whereas the active effect of VHL expression by ZBRK1 was largely attenuated by p300 depletion (Supplementary Figure S4F, S4G). These results suggested that ZBRK1 activates the transcription of VHL via p300.

VHL is a component of an E3 ubiquitin ligase and targets HIF1α for ubiquitylation and degradation. VHL also directly inhibits HIF1α transactivation by recruiting several proteins, including VHLaK [23]. To determine whether ZBRK1 interact with VHL/HIF complex and then suppress HIF transactivation, HEK293T cells was co-transfected with Flag-ZBRK1, HA-VHL, and EGFP-HIF1*α* and co-immunoprecipitation assay was then employed. Our results showed that HA-VHL co-precipitated efficiently with Flag-ZBRK1 but not EGFP-HIF1*α* (Supplementary Figure S5A). Given that p300 interacts with HIFα, we sought to determine whether the ZBRK1–p300 interaction dependent on HIFα. The ZBRK1-p300 interaction level was the same in HEK293T cells and HEK293T cells stably expressing *HIF1*α-shRNA and *HIF2*α-shRNA (Supplementary Figure S5B), suggesting ZBRK1 interacts with p300 independent of HIFα. Taken together, these results confirmed that VHL acts as an adaptor to bridge of ZBRK1 and p300.

**Figure 7:**
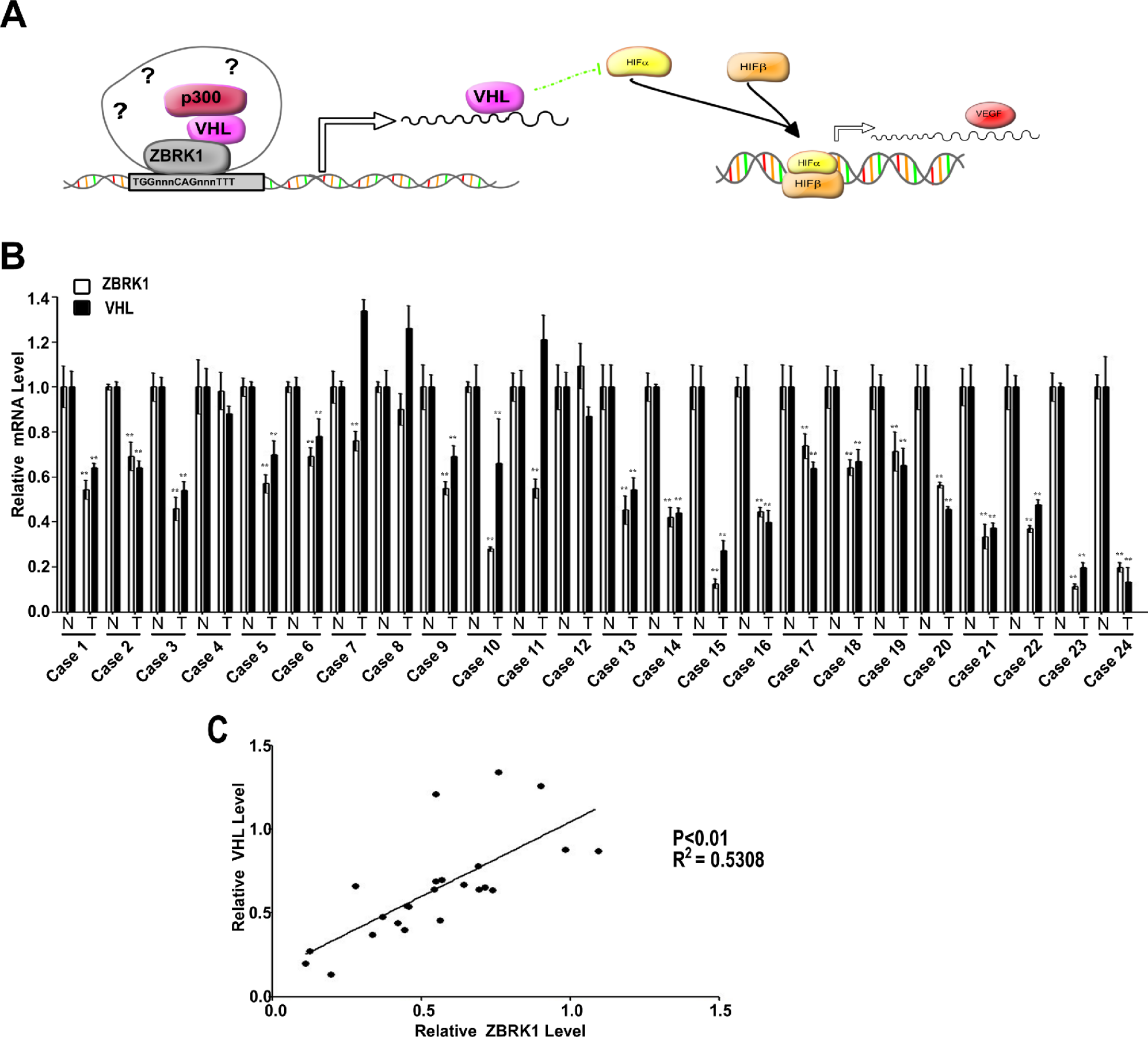
A schematic model for VHL and VEGF transcriptional regulation by ZBRK1 **(A)** ZBRK1, along with VHL and p300, forms an activator complex on VHL promoter via a ZBRK1 recognition element and activates transcription of VHL. The increased VHL expression promotes HIF-α ubiquitination and degradation. HIF-α, together with HIF-β, binds to promoter of downstream genes, and increases transcription of these genes such as VEGFα. **(B)** RT–qPCR analysis showed that VHL and ZBRK1 expression are reduced in ccRCC patient tissue. Bars indicate the standard error of the mean. **indicates significant differences, *P* < 0.01. **(C)** VHL correlates positively with ZBRK1expression (*P* < 0.01, R^2^ = 0.5308).

Finally, to demonstrate that ZBRK1/VHL/p300 complex is associated with VHL promoter in renal cancer cells, we performed a chromatin immunoprecipitation (ChIP) assay focusing on a 105 bp fragment around the ZBRK1 site (−2145/−2041). As shown in Figure 6H, we found DNA fragments containing the ZBRK1 site were highly enriched during immunoprecipitation with anti-ZBRK1, anti-VHL, and anti-p300 in ACHN cells. To verify the specificity of the precipitated 105 bp DNA fragment during our experiments, an additional qPCR amplification of a distinct genomic region (Exon 3) was performed on all of the precipitated chromatin DNAs, and we did not observe any enrichment of this DNA region as expected (Figure 6H). Taken together, our data presented here demonstrated that ZBRK1 binds to the VHL promoter via a ZBRK1 recognition element, and that p300 and VHL represents a co-activator regulating VHL expression through forming a complex with ZBRK1 (Figure 7A).

## DISCUSSION

In this study, our results demonstrated that VHL and p300, along with ZBRK1, form an activator complex on VHL promoter via a ZBRK1 recognition element (Figure 7A), and RT–qPCR analysis demonstrated that 22 of 24 renal cancer specimens expressed significantly reduced amounts of ZBRK1 RNA when compared with the normal tissues, among these, 19 of 22 renal cancer specimens expressed significantly reduced amounts of VHL expression (Figure 7B). Moreover, a significant positive correlation was found between VHL and ZBRK1 expression (Figure 7C). This result suggests that VHL gene transcription is partially correlates with low ZBRK1 transcripts in renal cancer patient tissue. Results from previous studies suggested that ZBRK1 may recruit co-regulators to form complex for its transcriptional regulation on gene expression [14], and these co-regulator, such as BRCA1 and Ataxin-2, may play important roles on the specificity of gene regulations [11, 12, 16, 24]. Our data showed that ZBRK1 interacts with the N terminal region of the VHL β domain through its KRAB and CTRD domains. Of note, the KRAB and CTRD domains are transcriptional repression domains located in the N- and C-terminal of ZBRK1, respectively [11]. The KRAB domain silences gene expression via recruitment of transcriptional repressor including KAP-1/KRIP-1/TIFb co-repressor [15, 21] and CTRD domain has been shown to be responsible for recruiting both homotypic and heterotypic proteins on ZBRK1 DNA consensus motifs and thus facilitating ZBRK1-directed transcriptional regulation [25]. Besides its canonical function as E3-ubiquitin ligase, VHL also behaves as a cofactor of p53 by recruiting p300 [22]. Based on our finding, we suggest that VHL may acts as a novel co-regulator of ZBRK1 through its binding to the CTRD of ZBRK1.

We here identified a single ZBRK1 recognition site in the VHL promoter by luciferase reporter assays and ChIP experiments and demonstrated that ZBRK1 up-regulates the VHL expression. Interestingly, VHL enhances ZBRK1 transcriptional activity in VHL promoter, this lead to a hypothesis that VHL protein may be essential for ZBRK1-enhanced transcriptional activity in the VHL promoter, and knock-down of VHL expression may eliminate the transcriptional activity of ZBRK1 on VHL expression. Thus, our data presented here provides the first evidence that VHL is implicated in transcriptional activation itself via binding to ZBRK1. Furthermore, we found that ectopically expression of ZBRK1 in VHL^−/−^ 786-O cells did not cause any changes on VHL-promoter-driven luciferase activity. We also found that the regulatory effect of ZBRK1 on VEGF, a HIF target gene, is dependent on the VHL/HIF pathway.

Although ZBRK1 are thought to function mainly as a transcriptional repressor, a dual function in gene repression and activation has been reported recently. Lin *et al*. demonstrated that numerous genes was transcriptional activated by ZBRK1 [14], however, the mechanistic details of ZBRK1 as a transcriptional activator and its co-activators still remain unclear. p300 is a well known transcriptional co-activator for many transcription factors, for example, p53 and HIF1 [22, 26]. In this study, our results suggested that functions as a co-activator of ZBRK1. It was reported that ZBRK1 is rapidly degraded through the ubiquitin-proteasome pathway upon treatment with the DNA-damaging agents [27]. Thus, it is very likely that the post-translational modifications of ZBRK1 play an important role in modulating its transcriptional regulatory properties. Despite that VHL has E3-ubiquitin ligase activity, VHL has no effects on the stability of ZBRK1 protein *in vivo* (Supplementary Figure S6). However, it was reported that several modifications, such as phosphorylation and acetylation, have been shown to be regulated by VHL [22, 28]. In addition, Yang *et al*. (2007) also reported that VHL served as an adaptor and promoted the phosphorylation of the Card9 of ZBRK1 by recuiting CK2 [28]. In this study, we domenstrated that VHL protein bridges an association between the acetyltransferase p300 and ZBRK1 to activate its own promoter. It is very likely that p300 catalyzes the acetylation of ZBRK1 and up-regulates the transactivity of ZBRK1. The potential effects of posttranslational modification of ZBRK1 on the transcriptional activity are currently under investigation.

ZBRK1 has been shown to be downregulated in human carcinomas when compared with their expression in corresponding para-carcinoma tissues [14, 16–18]. We also verified a low abundance of *ZBRK1* transcripts in renal cancer tissues in comparison with matching adjacent normal renal tissues. Most importantly, we analyzed the data from 155 Chinese ccRCC patients, and found that the expression level of ZBRK1 is clearly correlated with the grade, stage and metastasis index of tumors. Together with our findings in this study, we confirmed that over-expression of ZBRK1 inhibited cancer cell growth, migration and invasion *in vitro* and tumorigenicity and metastasis *in vivo*, knockdown of ZBRK1 could enhance cancer cell growth, migration and invasion *in vitro*. Also, it suggested that ZBRK1 functions as a tumor suppressor in renal cancer development and progression, and may serve as a potential prognostic marker for ccRCC patients.

In summary, we discovered a novel ZBRK1/VHL/p300 complex that binds to the VHL promoter and activates the expression of VHL. Inactivation of ZBRK1 may be involved in renal cancer progression. Further studies for characterization of ZBRK1 and its association with VHL will be helpful for our understanding of its roles and molecular mechanisms in tumorigenesis and progression of ccRCC.

## MATERIALS AND METHODS

### Antibodies

Following antibodies were used in the experiments: anti-Flag (F3165) from Sigma–Aldrich; anti-GFP (11814460001), anti-Myc (11667149001) and anti-HA antibody (11583816001) from Roche Applied Science; anti-ZBRK1(ab77085) from Abcam; anti-VHL (#2738) from Cell Signaling Technology; anti-VHL (D-7) and anti-p300 (N-15) from Santa Cruz Biotechnology; anti-GAPDH (CW0100) purchased from Beijing CWBio; anti-CD31 (11265-1-AP) purchased from Proteintech. Goat anti-mouse IgG horseradish peroxidase (HRP)-linked whole antibody (SA1-74039) and goat anti-rabbit IgG horseradish peroxidase (HRP)-linked whole antibody (SA1-9510) purchased from Pierce Company (Rockford, IL, USA). FITC-labeled or Cy3-labeled goat anti-rabbit or mouse IgG purchased from CWBIO.

### Plasmid constructs

CMV-p300 plasmid was from addgene (Plasmid 10717). The Y2H bait plasmid (BD-VHL) was described earlier [29]. The human VHL gene cloned into pGEX-4T-1 (GST-VHL), psi-Flag-Strep II (SF-VHL) and pRK-HA (HA-VHL) were from Prof. Rongjia Zhou (Wuhan University, China). GST-VHL was PCR amplified using primers VHL-5′ and VHL-3′, digested by BamHI and XhoI, and ligated into psi-Flag-C1, psi-mCherry-C1 and pcDNA3-Myc to create Flag-VHL, Cherry-VHL and Myc-VHL, respectively. To obtain the VHL fragment consisting of residues 1 to 154, 155 to 213, and 115 to 213, Flag-VHL was PCR amplified using primers VHL-5′ and VHL-N-3′, VHL-C1-5′ and VHL-3′, VHL-C2-5′ and VHL-3′, respectively. The products were digested with BamHI and XhoI and ligated into psi-Flag-C1. VHL deletion mutant lacking 115–154 was generated by a two-step PCR-based mutagenesis procedure using Flag-VHL as the template. First-step PCR was used to amplify two partially overlapping fragments using primers VHL-dE2-5′ plus VHL-3′, and VHL-5′ plus VHL-dE2-3′. Both fragments were annealed and used as the template for second-step PCR. Second-step PCR with primers VHL-5′ and VHL-3′ was used to obtain the VHLΔ115–154 mutant. The resulting PCR product was cloned into psi-Flag-C1 to obtain the Flag-VHLΔ115–154. Human cDNA was PCR amplified using primers ZBRK1-5′ and -3′, digested by BamHI and XhoI, and ligated into psi-Flag-C1, psi-EGFP-myc-C1 and psi-HA-C1 to create Flag-ZBRK1, EGFP-myc-ZBRK1 and HA-ZBRK1, respectively. To obtain the ZBRK1 fragment consisting of residues 1 to 168, 1 to 439, 168 to 439, 425 to 532, and 80 to 439, Flag-ZBRK1 was PCR amplified using primers ZBRK1-5′ plus ZBRK1-N-3′, ZBRK1-5′ plus ZBRK1-ZN-3′, ZBRK1-ZN-5′ plus ZBRK1-ZN-3′, ZBRK1-C-5′ plus ZBRK1-3′, and ZBRK1-dK-5′ plus ZBRK1-ZN-3′, respectively. The products were digested with BamHI and XhoI and ligated into psi-Flag-C1. The VHL-luciferase (VHL-luc) plasmids were constructed by cloning the promoter region of human VHL into the pGL3-basic vector (Promega). To obtain the VHL promoter constructs A (−2731/+103) and B (−2087/+103), human genome DNA was PCR amplified using primers VHL-A-p5′ plus VHL-p3′ and VHL-B-p5′ plus VHL-p3′ respectively. The products were digested with MluI and XhoI and ligated into pGL3-basic. To obtain construct C, construct A was subjected to a two-step PCR-based mutagenesis procedure for point mutations of ZBRK1 binding sites. Primers VHL-A-p5′ plus VHL-C-3′ and VHL-C-p5′ plus VHL-p3′ was used to obtain two segment, and the annealed DNA was then used as a template with primers VHL-A-p5′ and VHL-p3′. The products were digested with *Mlu*I and *Xho*I and ligated into pGL3-basic. PCR conditions and primers for the creation of these constructs are provided in Supplementary Table S1. All constructs were verified by sequencing.

### RNA interference

Oligos coresponding to the target sequences were annealed and cloned into the HpaI and XhoI sites of the pSicoR plasmid (Addgene). The following target regions were chosen: ZBRK1-1#, GCTAACCATGAACGACTTCAT; ZBRK1-2#, GGAG AACAACTGTGGACAA; VHL, GATCTGGAAGACCA CCCAAAT; HIF1α, GTCTGCAACATGGAAGGTA; HIF2α, GACATGTCCACAGAGCGGGAC; p300, GCTCATCCAGCAGCAGCTTG; EGLN1, GTCTCTCTA TAACATCTGAG; KAP1, GGACCACCAGTACCAGT TC; BRCA1, GTAGCTGATGTATTGGACG.

### Cell culture and transfection

ACHN, 786-O, SN12PM6, Caki-1 and HEK293T cells were cultured in Dulbecco's modified Eagle's medium (Invitrogen) with 10% fetal bovine serum (HyClone) in the presence of 5% CO2 at 37°C in a humidified incubator (Thermo Fisher Scientific). All transfections were per-formed using Lipofectamine™2000 (Invitrogen, Carlsbad, CA, USA).

### Virus generation and infection

Lentiviral vectors were transfected into HEK293T cells in combination with lentiviral packaging vectors pRSV-Rev, pMD2.G, and pCMV-VSV-G using Lipofectamine™2000. After transfection for 48 h, supernatants were collected and filtered through a 0.4 μm filter, and used directly to infect ACHN cells.

### Real-time PCR

Total RNAs were extracted by Trizol (Invitrogen) and cDNAs were synthetized using Rever Ace qPCR RT Kit (TOYOBO). Real-time PCR was performed using SYBR Green Realtime PCR Master Mix (TOYOBO) and the Stratagene Mx3000P QPCR System (Agilent Technologies). Amplification conditions were as follows: 95°C for 15 s, 60°C for 15 s, 72°C for 45 s for 40 cycles in a 25 μl reaction mix containing 1 × SYBR Green. Primers for the reaction are provided in Supplementary Table S1.

### Luciferase activity assays

ACHN, SN12PM6, Caki-1 or 786-O cells were grown in 24-well plates to 70–80% confluence and transfected with various plasmid combinations. Approximately 36 h after transfection, the expression of Firefly and Renilla luciferase were measured by the Dual-Luciferase™ Reporter Assay system (Promega).

### Y2H analysis

Y2H screening was performed as described previously [30]. First, BD-VHL was chosen as the bait and transformed into yeast strain AH109. Next, these yeast colonies were collected and transformed with cDNA library (Clontech). Positive clones were identified using the SD-Leu-His-Ade selection plates and β-galactosidase assay. The positive plasmids (prey) were isolated and amplified in *E.coli* DH10b. The respective bait or pGBKT7 (control) and prey plasmids were retransformed into yeast to verify the interactions.

### GST-pull down and co-immunoprecipitation assays

GST-pull down was performed as described previously [31]. GST-VHL or GST was expressed in *E. coli* BL21 cells, respectively. *E. coli* cells were lysed in NETN buffer (50 mM Tris–HCl, pH 8.0, 0.15 M NaCl, 1 mM EDTA, and 0.5% NP-40) containing protease inhibitor cocktail (Roche). GST–VHL and GST (control) were purified with the glutathione-Sepharose 4B beads and then incubated with the lysates from Flag-ZBRK1-transfected HEK293T cells. After washing, bound proteins were detected using anti-Flag antibody.

To analyze protein interactions, co-immunoprecipitation experiments were performed using HEK293T, ACHN or 786-O cells. The cells were transfected with different combinations of expression vectors and lysed in NETN buffer. Specified antibody and Protein G Agarose (Roche) were incubated with the cell lysates for overnight at 4°C. The resins were washed four times with buffer NETN. After elution by 1X loading buffer, and heated at 95°C for 5 min, the bound proteins were analyzed through western blotting.

### Isolation of cytosolic and nuclear proteins

Cytosolic and nuclear proteins was isolated following the procedure previously described [32]: The cells were collected by centrifugation (1000 g, 5 min) and resuspended in 100 μL of buffer A (10 mM Tris-HCl pH 7.4, 10 mM NaCl, 5 mM MgCl2, 1 mM DTT, protease inhibitor cocktail) for 20 min on ice, followed by the addition of 5 μL buffer B (10 mM Tris-HCl pH 7.4, 10 mM NaCl, 5 mM MgCl2, 1 mM DTT, protease inhibitor cocktail, 10% Igepal CA-630). The lysates were vigorously mixed and centrifuged for 5 min (500 g, 4°C). The cytoplasmic proteins were present in the supernatant. To extract the nuclear proteins, the nuclear pellet was resuspended in 300 μL of buffer A for 10 min at 4°C. After vigorous mixing, the nuclear suspension was centrifuged for 5 min at 4°C.

### ELISA

VEGF protein in culture supernatants was examined using a human VEGF ELISA (#KHG0111; Invitrogen Corporation, Camarillo, CA) normalized to total protein content as measured by Bradford assay.

### Chromatin immunoprecipitation (ChIP)

Cells were crosslinked with 1% formaldehyde-PBS for 15 min at room temperature. The cells were scraped and washed twice with cold PBS. Then, the cells were lysed in 1 ml cold cell lysis buffer (10 mM Tris-Cl, pH 8.0, 10 mM NaCl, 3 mM MgCl2, 0.5% NP-40) supplemented with protease inhibitors (Roche) and incubated at 4°C for 5 min to allow the release of nuclei. Cell nuclei were lysed with lysis buffer (10 mM Tris–HCl, pH 8.0, 100 mM NaCl, 1 mM EDTA and 0.1% sodium deoxycholate) and sonicated to solubilize and shear crosslinked DNA. After centrifugation, the supernatant chromatin was immunoprecipitated with 10 μg of indicated antibody. The beads were washed five times with NETN buffer. The precipitated DNA was eluted by heating at 65°C and crosslinking was reversed by overnight incubation at 65°C. The ChIP DNA was subjected to real-time PCR amplification using the primers specific for the promoters of genes analyzed (Supplementary Table S1).

### Tandem affinity purification (TAP)

ACHN cells were infected with Lentiviral plasmids encoding SF-tagged (Strep II tag and Flag tag)-VHL to establish stable cell lines expressing SF-VHL proteins. Cells (~6 × 10^7^) were lysed in NETN buffer containing protease inhibitor cocktail (Roche). The cell lysates were centrifuged at 13,000 × g for 10 min at 4°C to remove debris and then incubated with Strep-Tactin Sepharose (IBA, Göttingen, Germany) for 4 h at 4°C. The complexes were washed three times with NETN buffer and then bound proteins were eluted with NETN buffer containing 2.5 mM desthiobiotin (Sigma-Aldrich). The eluents were incubated with anti-ZBRK1 antibody and Protein G Agarose for overnight at 4°C. After three washes, the immunocomplexes were analyzed by Western blot with the appropriate antibodies.

### Double immunofluorescence

For immunofluorescence experiments, Caki-1 cells were fixed with 4% paraformaldehyde, permeabilized with 0.1% Triton X-100 in PBS, and stained with the mixture of two primary antibodies (rabbit against human ZBRK1 (Abcam, ab77085) and mouse against human VHL (Santa Cruz, sc-55506) in 1% BSA in PBST in a humidified chamber for overnight at 4°C. After washing with PBS, the cells were subjected to the mixture of two secondary antibodies (Cy3-conjugated against mouse and FITC-conjugated against rabbit) in 1% BSA for 1 hr at room temperature in dark. The nuclei were stained with Hoechst33258. Images were taken with a confocal fluorescence microscope.

### Immunocytochemistry

Xenograft tumors derived from ACHN cells were embedded in OCT medium (Tissue Tek, Miles, Elkhart, IN, USA) and cut into a series of 6 mm sections with a cryostat (Leica, Bensheim, Germany). To determine immunocytochemical localization, the sections were fixed with 4% paraformaldehyde for 20 min and then permeabilized with 0.1% Triton X-100 in PBS for 30 min. After being blocked with 3% H_2_O_2_ and nonimmune rabbit serum, sections were incubated at room temperature with anti-CD31 antibody Then SABC and DAB were used for color visualization according to the manufacturer's instructions (Boster Company, China).

### Patients and tumor samples

Written informed consent was obtained from all patients and the study was approved by the Institutional Review Board of Huazhong University of Science and Technology, Tongji Medical College, Tongji Hospital. Patients with clear cell carcinoma of kidney who received nephrectomy or partial nephrectomy were included in the study. The clinical information was retrieved from the medical records.

### Colony formation and cell proliferation

ACHN, SN12PM6 and Caki-1 cells were infected with lentiviral for 72 hours, then cells were digested and transferred to 96 well micro-plates, replanting at a density of approximately 2000(ACHN)/3000(SN12PM6)/4000(Caki-1) cells per well 24 h, 48 h, 72 h, 96 h and 120 h after infection. All experiments were performed in triplicate. Cell proliferation was estimated using the Cell Counting Kit-8 (CCK-8) (Dojindo Laboratories, Kumamoto, Japan) according to manufacturer instructions. 72 hours following infection, cells were seeded in 6-well plates at 1,000 cells/well, for colony formation, cells were maintained in complete medium for two weeks at which point crystal violet was used for visualizing colonies. The complete medium was changed every 3 days.

### Cell invasion and migration assays

Migration and invasion assays were performed using uncoated and Matrigel™ coated Transwell^®^ inserts according to manufacturer instructions. A density about 1 × 10^5^ of ACHN cells or 5 × 10^4^ of SN12PM6 or 5 × 10^4^ Caki-1 were suspended and then seeded in the upper chambers of 24-well transwell plates with FBS-free medium. Culture medium containing 10% fetal bovine serum was deposited in the lower chambers. After 12–18 hours incubation at 37C in a humidified 5% CO2 atmosphere for ACHN, SN12PM6 and Cak-1, cells that migrated were stained by 0.5% crystal violet solution for 15 min and counted. For invasion, Transwell membranes were prepared with matrigel for plating infected cells. After 24 hours for ACHN, SN12PM6 and Caki-1, cells that invaded were stained by 0.5% crystal violet solution for 15 min and quantified by determining the total cell number derived from 5 randomly chosen visual fields per membrane at 400× magnification. Each experiment was performed in duplicate.

### *In vitro* tube formation assay

HUVECs were maintained in basic medium containing 2% FBS and 1% penicillin/streptomycin or the indicated conditioned media (CM) of ACHN, SN12PM6 and Caki-1 cells. HUVECs (6 × 10^4^) were seeded into a 48-well culture plate precoated with Matrigel (BD Biosciences) overnight and then cultured in the indicated condition. After 4–6 h incubation, the formation of tubes was photographed with a phase contrast microscopy (100 × magnification, Olympus Instruments, Inc.), and quantified by counting branch points in five randomly selected microscope fields per well. The experiments were conducted twice in duplicate.

### Animal experiments

Tumorgenesis in nude mice was determined as described previously [33]. Two groups of five mice each were injected subcutaneously with prepared cells at a single site. Tumor onset measured with calipers at the site of injection weekly at different times on the same day. Tumor volume was calculated using the formula, 0.5ab2, where a represent the larger and b represents the smaller of the two perpendicular indexes. Animals were sacrificed 34 days after injection. These tumors were weighed and verified by hematoxylin and eosin (H&E) staining. The vascularity evaluation was taken by immunohistochemical staining with CD31 antibody (Abcam). Nude mice were manipulated and cared for according to NIH Animal Care and Use Committee guidelines in the Experiment Animal Center of the Tongji medical collage of Huazhong University of science and technology.

### Metastasis assay

The antimetastatic activity of ZBRK1 was tested in the mouse ACHN lung metastasis model as described previously [29]. ACHN cell was stably infected with this gene containing GFP label. Treated cells (2 × 10^5^) were suspended in 100 μL of PBS and injected intravenously via the tail vein. Mice were sacrificed and lungs were resected 30 days later after injection. The incidence and volume of metastases were estimated by imaging of mice for bioluminescence using the Living Image software (Xenogen, Baltimore, MD). The photon emission level was used assess the relative tumor burden in the mice lungs. All animal studies were conducted under approved guidelines of the Animal Care and Use Committee of the Tongji Hospital (Wuhan, China).

### Statistical analysis

The data are presented as the mean ± SEM. Differences among groups were determined by a two-way ANOVA followed by a post hoc Tukey test. Comparisons between two groups were performed using an unpaired Student's *t* test. A value of *P* < 0.05 was considered significant.

## SUPPLEMENTARY FIGURES AND TABLE


