# Supplementary material for: ZBRK1, a novel tumor suppressor, activates VHL gene transcription through formation of a complex with VHL and p300 in renal cancer
Source: Oncotarget. 2015 Feb 10;6(9):6959–76. doi: 10.18632/oncotarget.3134 (PMC4466662; doi:10.18632/oncotarget.3134)
Supplement: Supplementary file 1 [file oncotarget-06-6959-s001.pdf]

SUPPLEMENTARY FIGURES AND TABLE

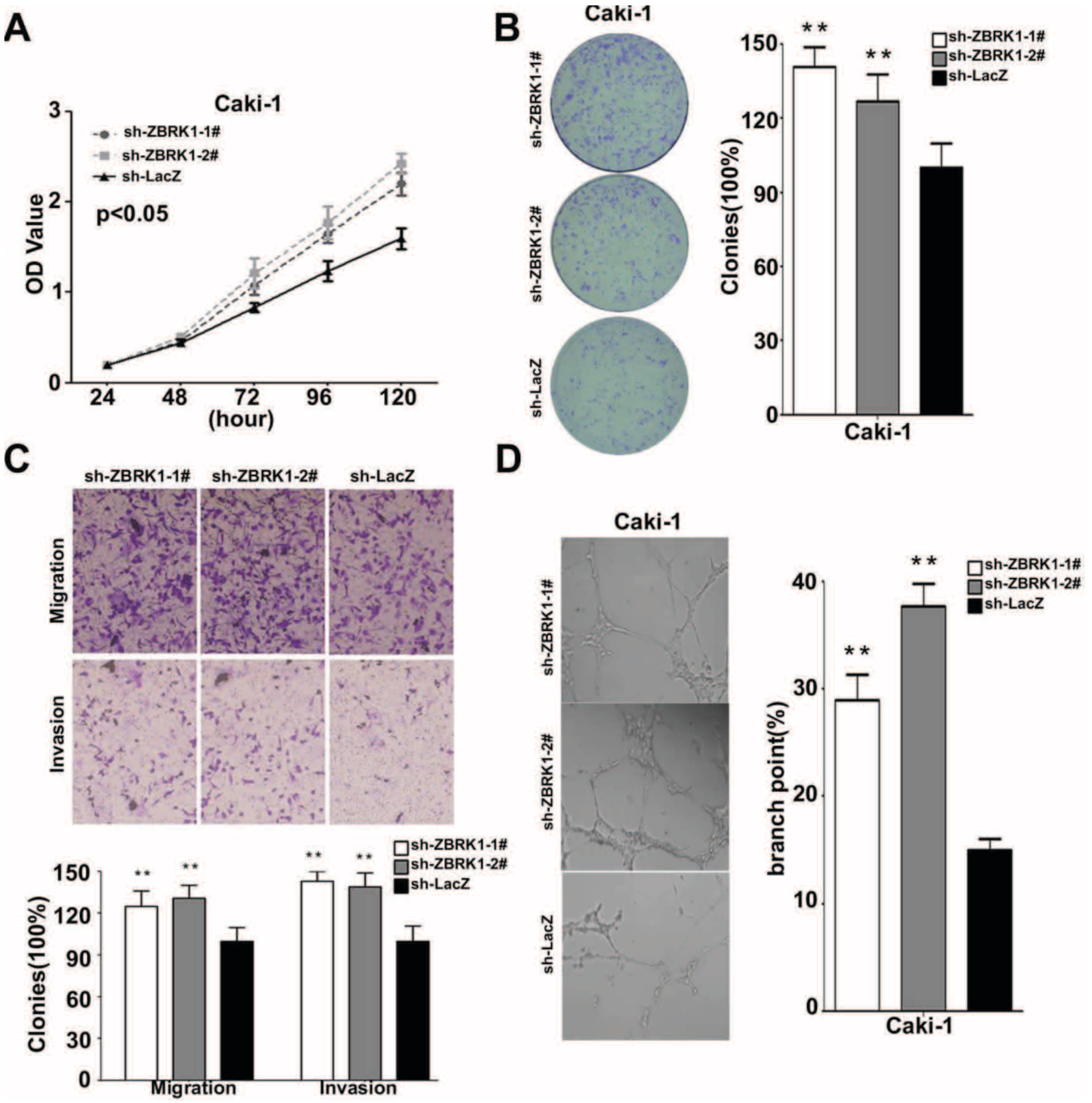

Supplementary Figure S1: Knockdown of ZBRK1 enhance cell growth, tube formation, migration and invasion in renal cancer. (A) CCK-8 kit was utilized to quantify cell viability at each time point. Data are plotted as the mean ± SEM of 3 independent experiments. **(B)** a'. Representative photographs of cell culture plates following staining for colony formation of Caki-1 cells. b'. Number of colonies was quantified. **(C)** a'. Migration and invasion assay for renal cancer cells. Representative photographs were taken at × 200 magnification. b'. Number of migrated and invaded cells were quantified in 4 random images from each treatment group. Results are the mean ± SEM from 3 independent experiments plotted as percent (%) migrating and invading cells relative to NC treatment. **indicates significant differences, $P < 0.01$. **(D)** a'. Tube formation of HUVECs was determined by assaying the numbers of branch nodes after 6 h of culture under a phase contrast microscope. HUVECs were cultured in the following media: CM of Caki-1 cells transfected with negative control and CM of Caki-1 cells infected with knockdown constructs (lentiviral transfer of ZBRK1-specific shRNA). b', number of branch point was quantified.

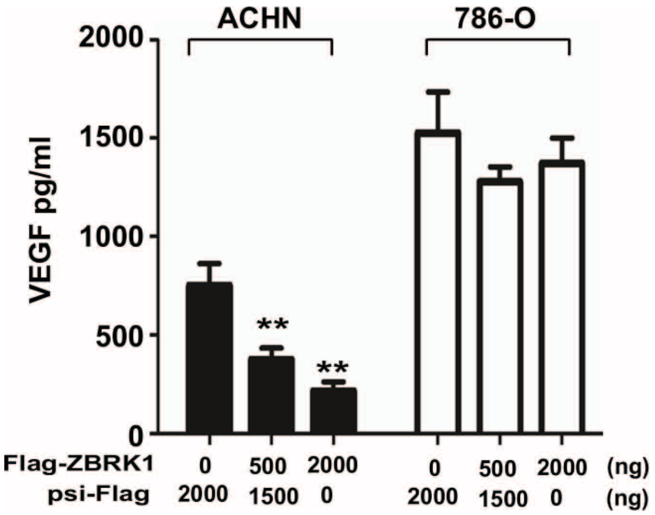

Supplementary Figure S2: VEGF expression in culture supernatants of ACHN cells but not 786-O cells. ACHN cells or 786-O cells were transfected with increasing amount of Flag-ZBRK1. After transfection for 48 h, VEGF in the culture supernatants was determined by ELISA as described in Materials and methods. VEGF values were normalized to total cellular protein. Means ± standard deviations for *n* = 3 are shown. ***p* < 0.01 vs. Ctrl.

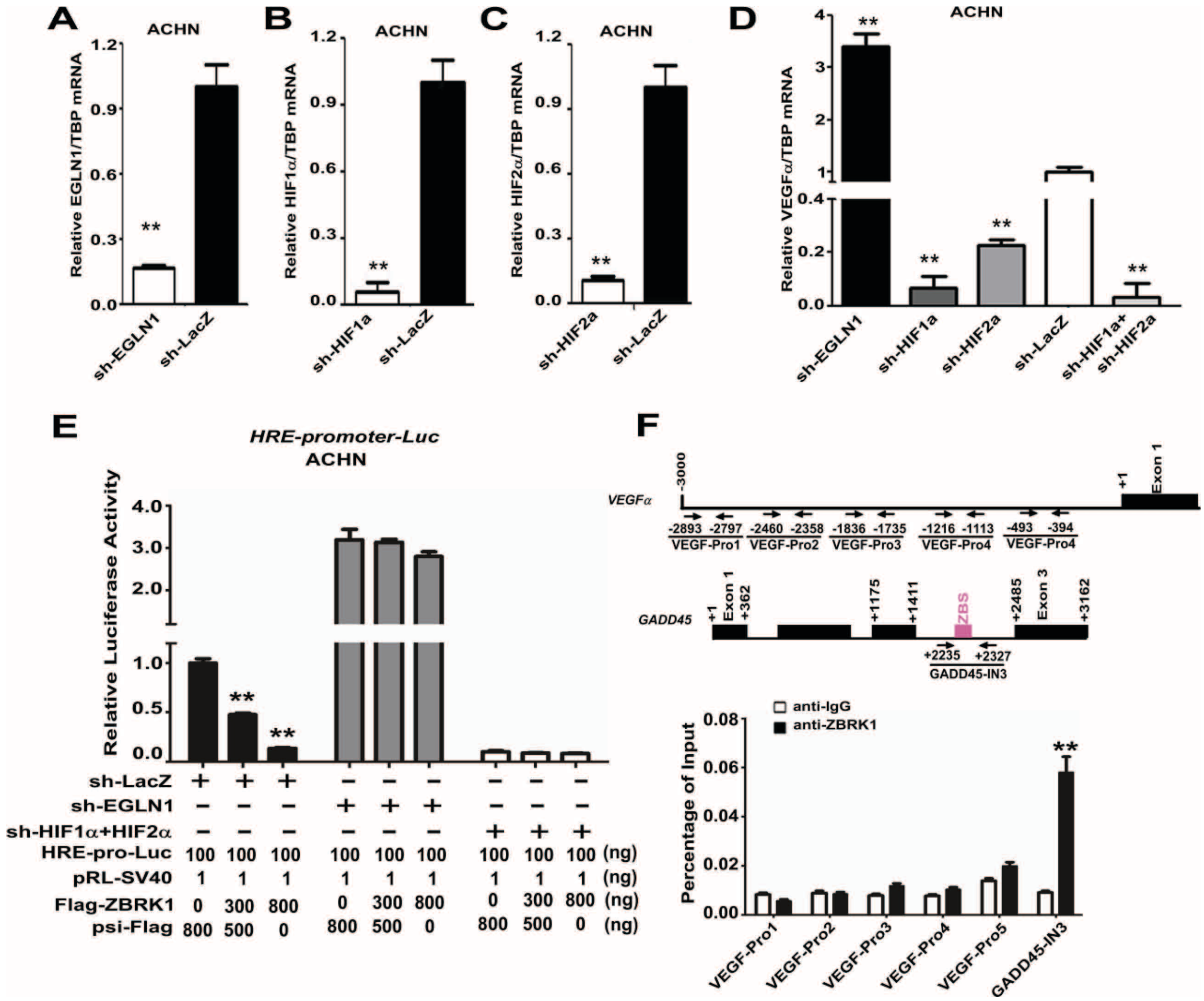

Supplementary Figure S3: ZBRK1 inhibits the expression of VEGF dependent on EGLN1/HIF/VHL pathway. **(A–D)** Total RNAs were extracted from ACHN cells stably expressing *EGLN1*-specific shRNA, *HIF1*α-specific shRNA and/or *HIF2α*-specific shRNA, followed by Real-time PCR using specific primers for EGLN1 (A), HIF1α (B), HIF2α (C), VEGFα (D), and TBP (inner control). Bars indicate the standard error of the mean. $^{**}p < 0.01$ vs. sh-LacZ. **(E)** HIF is required for ZBRK1 mediated inhibition of HRE-promoter-driven luciferase activity. ACHN cells stably expressing LacZ-shRNA, EGLN1 shRNA, or HIF1α and HIF2α shRNA were transiently transfected with 6xHRE-promoter-driven luciferase reporter genes and pRL-SV40 along with increasing amount of Flag-ZBRK1. After transfection for 36 H, luciferase activities were measured and normalized with inner control. $^{**}p < 0.01$ vs. Ctrl. **(F)** ZBRK1 can not binds to the VEGF promoter. ChIP analysis on five fragments in the *VEGFα* promoter and a fragment in *GADD45* Intron 3 (containing a ZBRK1 recognition sequence) to detect the association of ZBRK1 in ACHN cells. The cells were lyzed and chromatin DNA was immunoprecipitated with anti-ZBRK1 validated by qPCR. Means ± standard deviations for $n = 3$ are shown. $^{**}p < 0.01$ vs. anti-ZBRK1/GADD45-IN3.

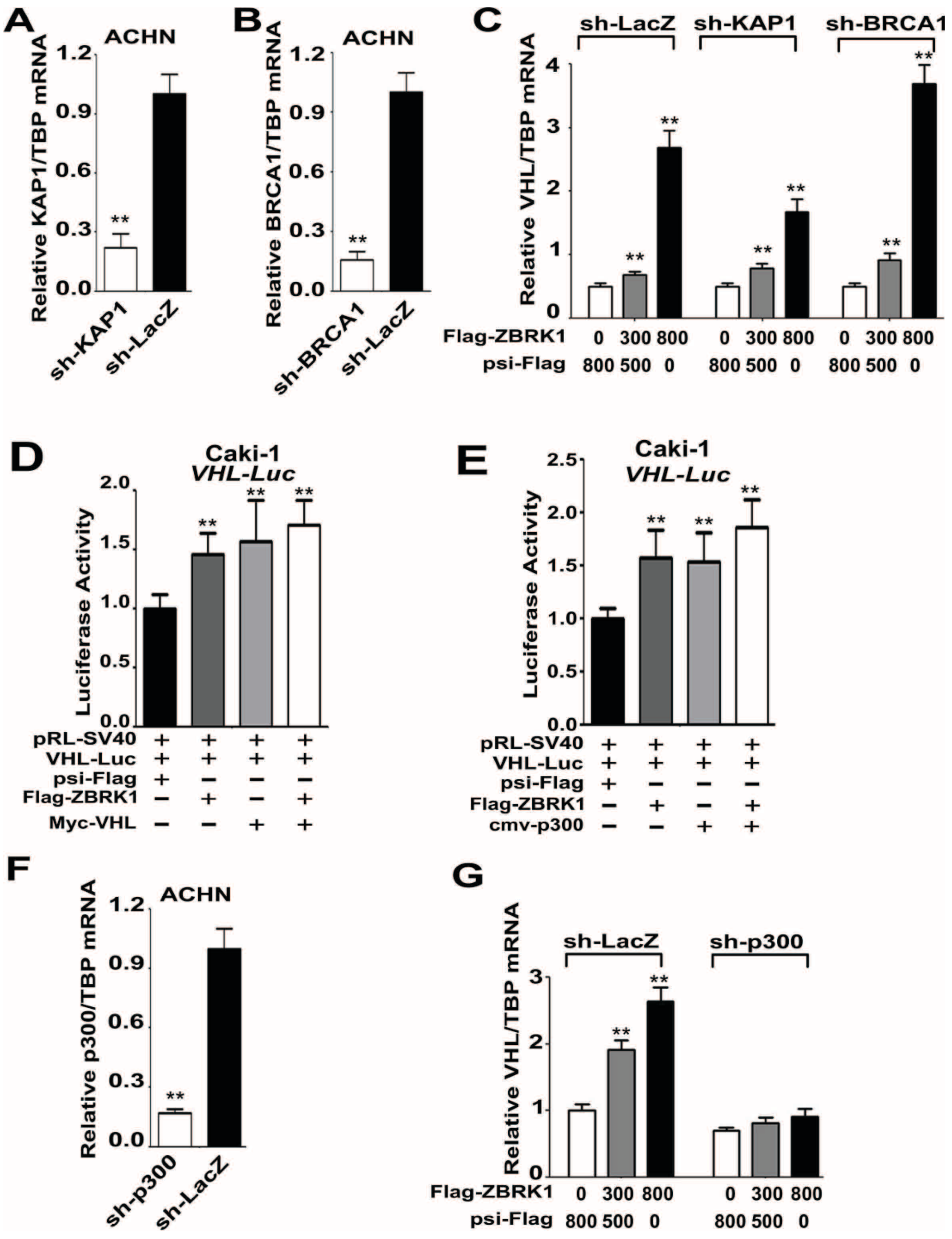

Supplementary Figure S4: ZBRK1 activates the transcription of VHL via p300, but not KAP1 and BRCA1. **(A)** qPCR analysis of KAP1 in ACHN cell expression expressing *KAP1*-shRNA and *LacZ*-shRNA. **(B)** qPCR analysis of BRCA1 in ACHN cell expressing *BRCA1*-shRNA and *LacZ*-shRNA. **(C)** ACHN cells expressing *LacZ*-shRNA, *KAP1*-shRNA, or *BRCA1*-shRNA were transfected with increasing amount of Flag-ZBRK1. Total RNAs were prepared and Real-time PCR was carried out using specific primers for VHL and TBP (inner control). **(D, E)** VHL and p300 enhances ZBRK1 transcriptional activity on VHL promoter in Caki-1 cells. Caki-1 cells were transiently transfected with different combinations of expression vectors for ZBRK1, VHL, and/or p300 along with pSL-SV40 and VHL-promoter driven luciferase reporter gene, luciferase activities were measured and normalized with inner control. Means ± standard deviations for $n = 3$ are shown, **indicates significant differences, $P < 0.01$. **(F)** qPCR analysis of p300 in ACHN cell expression expressing *p300*-shRNA and *LacZ*-shRNA. **indicates significant differences, $P < 0.01$. **(G)** qPCR analysis of VHL in ACHN cell expression expressing *p300*-shRNA or *LacZ*-shRNA which transfected with increasing amount of Flag-ZBRK1. Data are plotted as the mean ± SD of 3 independent experiments, **indicates significant differences, $P < 0.01$.

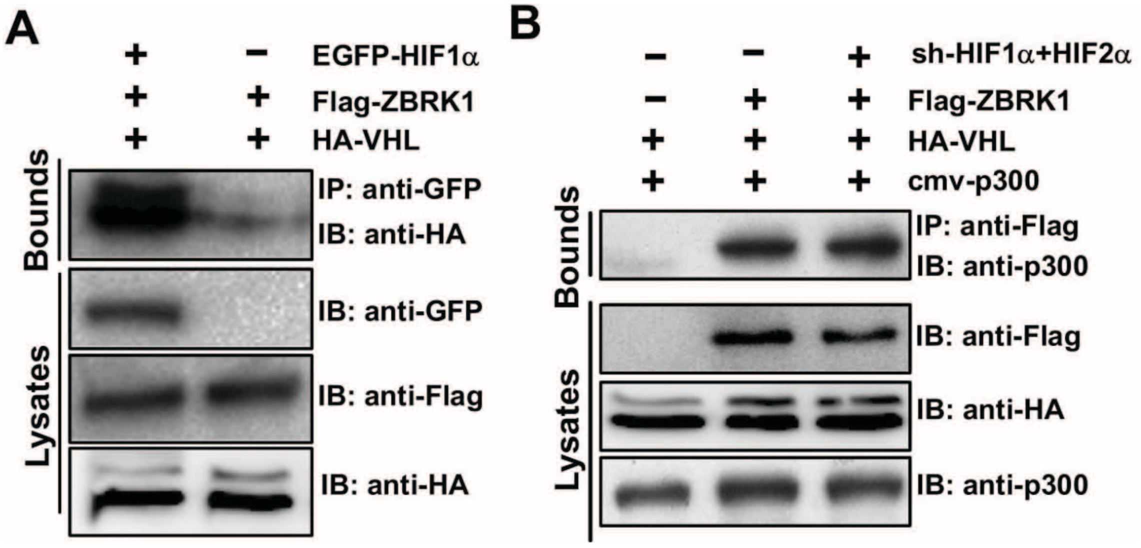

Supplementary Figure S5: ZBRK1 interacts with p300 independent on HIFα. **(A)** ZBRK1 cannot interaction with HIF1α. 293T cells were transiently transfected with Flag-ZBRK1, HA-VHL, or/and EGFP-HIF1α. The cell lysates were precipitated with anti-GFP antibody and immunoblotted with anti-Flag (no band) and anti-HA antibody. **(B)** The ZBRK1-p300 interaction levels not change when HIFα depletion. 293T cells and 293T cells stably expressing *HIF1α*-shRNA and *HIF2α*-shRNA were transfected with Flag-ZBRK1, HA-VHL, and cmv-p300. The cell lysates were immunoprecipitated with anti-Flag and immunoblotted with anti-p300.

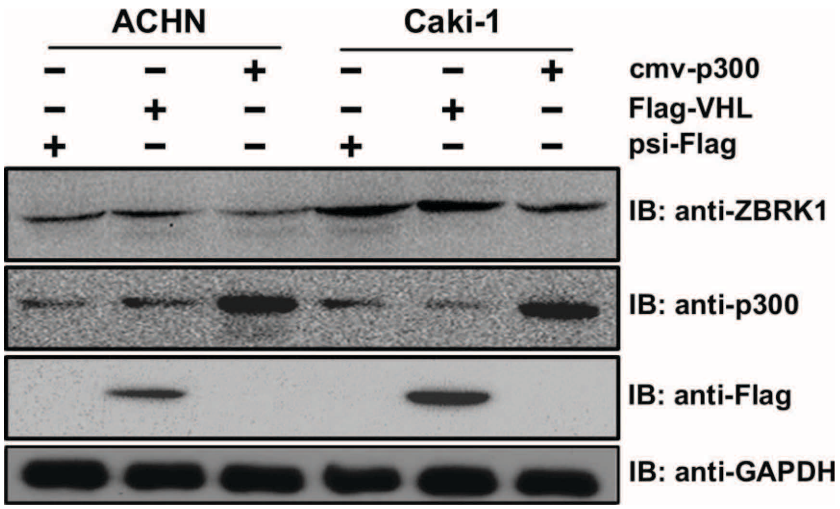

Supplementary Figure S6: VHL and p300 have no effect on the stability of ZBRK1 protein. ACHN and Caki-1 cells were transiently transfected with Flag-VHL or cmv-p300. Total protein was prepared from the cells and immunoblots were carried out using anti-ZBRK1, anti-VHL, anti-p300, or anti-GAPDH.

Supplementary Table S1: The primers used in the study Sites for restriction enzymes are underlined

Primer name	Primer sequence (5′-3′)	PCR
**Primers for making constructs**		
VHL-5’	CGCGGATCCGAATTCATGCCCCGGAGGGCGGAGAAC	94°C, 30 s; 58°C, 30 s; 72°C, 30 s; 35 cycles
VHL-3’	CCGCTCGAGTCAATCTCCCATCCGTTGATG	
VHL-N-3’	CCGCTCGAGTCATGGCAGTGTGATATTGGCAAAAAT	
VHL-C1–5’	CGCGGATCCGTGTATACTCTGAAAGAGC	
VHL-C2–5’	CGCGGATCCCACCTTTGGCTCTTCAGAG	
VHL-dE2–5’	CACAGCTACCGAGGTGTGTATACTCTGAAAGAG	
VHL-dE2–3’	GAGTATACACACCTCGGTAGCTGTGGATGCGG	
ZBRK1–5’	CGCGGATCCATGATCCAGGCCCAGGAATC	94°C, 30 s; 58°C, 30 s; 72°C, 1 min 50 s; 25 cycles
ZBRK1–3’	CCGCTCGAGCTAGTTAGCATGAAGAAAGGAGTC	
ZBRK1-N-3’	CCGCTCGAGTCAGAAAGCCCCGATTTCAGT	
ZBRK1-ZN-5’	CGCGGATCCAACCATGAACGACTTCATACT	
ZBRK1-ZN-3’	CCGCTCGAGCTATGCAGGAGGATTTTCCACCT	
ZBRK1-C-5’	CGCGGATCCACAAGGGAGAAACAAGAGGCA	
ZBRK1-dK-5’	CGCGGATCCGACATATGGAAAGTTGATCATG	
VHL-A-p5’	CCGACGCGTGTTTCATTTTAGGGATGTAGGCA	94°C, 30 s; 60°C, 30 s; 72°C, 2 min 30 s; 40 cycles
VHL-p3’	CCGCTCGAGCTCGGTAGAGGATGGAACGC	
VHL-B-p5’	CCGACGCGTGTGGAGATATCCAACTCCTG	
VHL-C-5’	AGGTTCATTTTGTTTGTGCTTTTGTGGAGATGTCCA	
VHL-C-3’	AAAAGCACAAACAAAATGAACCTAAGTAATTTTGCCT	
**Primers for real-time quantitative PCR analysis**		
TBP	F: TGCACAGGAGCCAAGAGTGAA	95°C, 15 s; 60°C, 15 s; 72°C, 45 s; 40 cycles
	R: CACATCACAGCTCCCCACCA	
ZBRK1	F: GACATATGGAAAGTTGATCATGTGCTG	95°C, 15 s; 60°C, 15 s; 72°C, 45 s; 40 cycles
	R: ATTCACTGCACACATGATGCTTCTCTA	
VEGFα	F: ACCTCCACCATGCCAAGTG	95°C, 15 s; 60°C, 15 s; 72°C, 45 s; 40 cycles
	R: TCTCGATTGGATGGCAGTAG	
KAP1	F: GGAAGGCTATGGCTTTGGG	95°C, 15 s; 60°C, 15 s; 72°C, 45 s; 40 cycles
	R: CCAGGCGTTCAAGGCTCA	

(*Continued*)

Primer name	Primer sequence (5′-3′)	PCR
BRCA1	F: GGGCTGGAAGTAAGGAAACAT	95°C, 15 s; 60°C, 15 s; 72°C, 45 s; 40 cycles
	R: ATGCTGCTATTTAGTGTTATCC	
p300	F: AGCAAAGAACCAGCAGAATCA	95°C, 15 s; 60°C, 15 s; 72°C, 45 s; 40 cycles
	R: GCGTTTGAACTCCTACACCTC	
HIF1α	F: GCAAGCCCTGAAAGCG	95°C, 15 s; 60°C, 15 s; 72°C, 45 s; 40 cycles
	R: GGCTGTCCGACTTTGA	
HIF2α	F: GTCTCTCCACCCCATGTCTC	95°C, 15 s; 60°C, 15 s; 72°C, 45 s; 40 cycles
	R: GGTTCTTCATCCGTTTCCAC	
VHL	F: ATGGCTCAACTTCGACGGC	95°C, 15 s; 60°C, 15 s; 72°C, 45 s; 40 cycles
	R: CCAGAAGCCCATCGTGTGTC	
VHL-Promoter	F: TGAGCTGTCAAGGAGAGCCC	95°C, 15 s; 60°C, 15 s; 72°C, 45 s; 40 cycles
	R: GGAGAAAATGACAGTATGAGTGAAC	
VHL-Exon3	F: GGTATTTCTTGGCATGCATCTT	95°C, 15 s; 60°C, 15 s; 72°C, 45 s; 40 cycles
	R: CAATCCTCTTACCTTGGCTTTC	
VEGF-Promoter1	F: GTGGGCTGCTCTCTGCTTCA	95°C, 15 s; 60°C, 15 s; 72°C, 45 s; 40 cycles
	R: CCCCAAAGAATCCTCCGAAG	
VEGF-Promoter2	F: AGGTGGGCCATACTTTCTCG	95°C, 15 s; 60°C, 15 s; 72°C, 45 s; 40 cycles
	R: GGGGAGTGAGGGCACAGAAT	
VEGF-Promoter3	F: GAGACAGGACTAGTGCACGA	95°C, 15 s; 60°C, 15 s; 72°C, 45 s; 40 cycles
	R: CAGTTCCATCGGTATGGTGT	
VEGF-Promoter4	F: TTGGGCTGATAGAAGCCTTG	95°C, 15 s; 60°C, 15 s; 72°C, 45 s; 40 cycles
	R: GCCTGCGTGATGATTCAAAC	
VEGF-Promoter5	F: GGGTGAGTGAGTGTGTGCGT	95°C, 15 s; 60°C, 15 s; 72°C, 45 s; 40 cycles
	R: TGGGGAGAGGGACACACAGA	
GADD45-Intron3	F: ATATGTATGTGGTTCAGCTTGG	95°C, 15 s; 60°C, 15 s; 72°C, 45 s; 40 cycles
	R: GGGTTCCCTGCAAAAGCGTT	
